# Comprehensive analysis of prognostic gene signatures based on immune infiltration of ovarian cancer

**DOI:** 10.1186/s12885-020-07695-3

**Published:** 2020-12-07

**Authors:** Shibai Yan, Juntao Fang, Yongcai Chen, Yong Xie, Siyou Zhang, Xiaohui Zhu, Feng Fang

**Affiliations:** 1 Department of Medical Oncology, the First Affiliated Hospital of Guangxi Medical University, Nanning, 530021 Guangxi Zhuang Autonomous Region China; 2 Laboratory of Experimental Cardiology, Department of Cardiology, University Medical Center Utrecht, Utrecht, 3584 CX The Netherlands; 3 Department of Obstetrics and Gynecology, The First People’s Hospital of Foshan, 81 Lingnan North Avenue, Foshan, 528000 Guangdong China; 4 Department of Pharmacology, College of Pharmacy, Shenzhen Technology University, Shenzhen, 518118 Guangdong China

**Keywords:** Ovarian cancer (OV), Single-sample gene set enrichment analysis (ssGSEA), Immune infiltration, Prognosis

## Abstract

**Background:**

Ovarian cancer (OV) is one of the most common malignant tumors of gynecology oncology. The lack of effective early diagnosis methods and treatment strategies result in a low five-year survival rate. Also, immunotherapy plays an important auxiliary role in the treatment of advanced OV patient, so it is of great significance to find out effective immune-related tumor markers for the diagnosis and treatment of OV.

**Methods:**

Based on the consensus clustering analysis of single-sample gene set enrichment analysis (ssGSEA) score transformed via The Cancer Genome Atlas (TCGA) mRNA profile, we obtained two groups with high and low levels of immune infiltration. Multiple machine learning methods were conducted to explore prognostic genes associated with immune infiltration. Simultaneously, the correlation between the expression of mark genes and immune cells components was explored.

**Results:**

A prognostic classifier including 5 genes (CXCL11, S1PR4, TNFRSF17, FPR1 and DHRS95) was established and its robust efficacy for predicting overall survival was validated via 1129 OV samples. Some significant variations of copy number on gene loci were found between two risk groups and it showed that patients with fine chemosensitivity has lower risk score than patient with poor chemosensitivity (*P* = 0.013). The high and low-risk groups showed significantly different distribution (*P* < 0.001) of five immune cells (Monocytes, Macrophages M1, Macrophages M2, T cells CD4 menory and T cells CD8).

**Conclusion:**

The present study identified five prognostic genes associated with immune infiltration of OV, which may provide some potential clinical implications for OV treatment.

**Supplementary Information:**

The online version contains supplementary material available at 10.1186/s12885-020-07695-3.

## Background

Ovarian cancer (OV), a highly malignant gynecologic tumour, is the leading cause of cancer-related mortality in women, and lack of specific symptoms at the early stage. Despite aggressive frontline treated with surgery and adjuvant chemotherapy, the overall survival rate of 5 years is still about 30% for most women diagnosed with advanced stages III/IV disease [1–3]. Tumour microenvironment (TME) is the primary or metastatic niche, in which tumour cells cooperate with the host stroma, such as various immune cells, endothelial cells, fibroblasts and metabolites. Recently, TME is playing an increasingly important role in the beginning and development of OV as well as anti-tumour treatment [4]. Immune system has also been reported as a critical factor in the initiation and development of cancer. Actually, many studies have confirmed that immune cells and immune-related genes (IRGs) are tempting targets for regulating tumour progression [5, 6]. The extent of immune cell infiltration is associated with clinical treatments and prognostic outcomes in OV patients, finding factors that drive infiltration will be the key to reveal outcome heterogeneity in this cancer [7, 8]. Numerous evidences support that OV is an immunogenic tumour [9, 10] and immunotherapy is an efficient strategy due to its highly targeted on the immune checkpoints [5, 11]. Besides, the prognostic assessment of immune system in OV has already been verified by previous researches [12–14]. Thus, it is pivotal to find out immune-related prognostic features in the treatment of OV.

With the development of human gene sequencing technology, high-throughput gene expression profiles have been widely used to detect the biomarkers of human diseases, which provides a chance to explore effective indicators for guiding the diagnosis, treatment and evaluating the prognosis of ovarian cancer [15, 16]. In recent years, database-based bioinformatic analysis of expression profile has been widely applied to screen out target biomarkers of diagnostic and prognostic value [17]. For example, Li et al. performed a series of analyses to identify four immune-related genes as biomarkers correlated with breast cancer prognosis. Shen et al. developed a prognostic signature which could be used to predict ovarian cancer survival [18, 19]. In addition, the emergence of genetic biomarkers contributes to adjusting treatment strategy and reducing unnecessary treatments. The public databases with complete gene expression profile and clinical information offer an opportunity for identifying immune-relevant prognostic features in OV.

In this study, our aim was to establish and validate an individualized prognostic gene signature for OV, which would evaluate the association between immune infiltration and the prognosis of OV.

## Methods

### Data collection and preprocessing

In this study, gene expression profile and relevant clinical information of 365 OV patients were downloaded from The Cancer Genome Atlas (TCGA) data portal (https://portal.gdc.cancer.gov/) and used as the research object. The primary clinical information in this database includes age, gender, race, smoking history, FIGO stage, survival status and survival time. To further utilize the large data sets of different genome libraries for verification, the standardized expression profile of mRNA-seq from International Cancer Genome Consortium (ICGC, https://icgc.org) and microarray matrix transformed by GPL96 and GPL14951 platform from Gene Expression Omnibus (GEO, https://www.ncbi.nlm.nih.gov/geo/) was acquired to conduct survival analysis as the validation set 1 (ICGC-OV-AU), validation set 2 (GSE14764, GSE23554 and GSE26712) [20–22] and validation set 3 (GSE140082; https://www.ncbi.nlm.nih.gov/geo/query/acc.cgi). Among them, validation set 2 combined three independent datasets adjusted by ComBat method from R package “sva” (version 3.38.0; https://bioconductor.org/packages/release/bioc/html/sva.html) [23]. According to the clinical information of all the OV patients, the patients with loss of survival time or less than 30 days were deleted at the beginning of this study to eliminate the interference of nonneoplastic factors. In the end, a total of 1129 patients with OV were enrolled in this study for further analysis.

### Clustering based on single-sample gene set enrichment analysis (ssGSEA)

The ssGSEA [24], an extension of Gene Set Enrichment Analysis, calculates separate enrichment scores for each pairing of a sample and gene set. Each ssGSEA enrichment score represents the degree to which the genes in a particular gene set are coordinately up or down-regulated within a sample. The method was used to quantify the activity or enrichment level of 29 immune-related gene sets representing different immune cell types, functions and pathways of each sample in TCGA-OV cohort. Characterized by the ssGSEA score, R package ConsensusClusterPlus (version 1.54.0; http://www.bioconductor.org/packages/release/bioc/html/ConsensusClusterPlus.html) [25] was applied to divide the samples into two categories with different levels of immune infiltrates. Considering the complex composition of tumour microenvironment including stromal cells, inflammatory cells, vascular system and extracellular matrix, R package ESTIMATE (version 2.0.0; https://bioinformatics.mdanderson.org/estimate/rpackage.html) [26] was used to evaluate the tumour purity and the number of stromal cells and immune cells that make up the major non-tumour components in tissues.

### Differential analysis of gene expression and support vector machine-recursive feature elimination (SVM-RFE)

To exclude genes not significantly related to immune cells infiltration in the tumour microenvironment, an analysis was performed via limma package (version 3.44.3; http://www.bioconductor.org/packages/release/bioc/html/limma.html) to preserve significantly differential expression genes (DEGs) between tumour and non-tumour components while |log2 foldchange| > 1 and FDR < 0.05 were considered as significant. Support vector machine is a classifier that can maximize the interval between categories. It can map the data to high dimensional space and realize linear separability of the data [27]. In this study, to get genes as the optimal feature to distinguish two different standards of immune infiltration of tumour sample, SVM function in e1071 package (Version: 1.7–4; https://cran.r-project.org/web/packages/e1071/index.html) of R was trained with a 5-fold cross-validation method followed by establishing accuracy and error [28].

### Weighted gene co-expression network analysis (WGCNA)

WGCNA (version 1.69; https://cran.r-project.org/web/packages/WGCNA/index.html) is a tool used to find out the module of co-expression genes, and to explore the relationship between gene network and phenotypes, as well as the core genes in the network [29]. We combined gene expression profiles with clinical information (overall survival time, overall survival status and FIGO stage) to screen samples for the co-expression module. Measurement of gene significance and module membership was utilized to discern the gene modules which closely connected with clinical characteristics. Ultimately, the integrated outcome of DEGs, SVM-RFE and WGCNA was embedded into next step analysis.

### Survival analysis

To filter mRNA related to the prognosis of ovarian cancer, univariate Cox regression analysis was performed for the genes in prognosis-related modules via the R package “survival” (version 2.41–3; https://cran.r-project.org/web/packages/survival/index.html), and *P* < 0.05 was considered statistically significant. Least absolute shrinkage and selection operator (LASSO) analysis (glmnet package, version 3.0–1; https://cran.r-project.org/web/packages/glmnet/index.html) was used to make further effort to screen out the key mRNA affecting the prognosis of OV by adjusting the regression coefficient and avoid the risk of over-fitting. For constructing a prognostic model, the key mRNA screened by LASSO regression analysis was further calculated by multivariate Cox regression analysis and the risk score of each patient was calculated. The risk score of each ovarian cancer sample in the prediction model is based on the following formula: Risk score = βgene (1) × exprgene (1) + βgene (2) × exprgene (2) + ··· + βgene(n) × exprgene(n). Among them, βgene refers to the coefficient of each gene in the multivariate Cox regression analysis, and exprgene represents the expression level of each gene. The cut-off point of risk value which is most related to survival is determined by the surv_cutpoint function in the survminer R package (version 0.4.3; http://www.sthda.com/english/wiki/survminer-r-package-survival-data-analysis-and-visualization), which regarded as the standard to separate the high- and low-risk groups in the OV cohort.

### Comparison of tumour mutation burden (TMB), DNA damage repair (DDR) and copy number variation (CNV) levels between the subgroups

TMB refers to the total number of mutations per megabyte of base sequence in the exon coding region of the evaluated gene in a tumour sample [30], which is closely related to the effect of immunotherapy [31, 32]. TCGA-OV masked somatic mutation in MAF format was downloaded and oncolot function in R package maptools (version 1.0–2; https://cran.r-project.org/web/checks/check_results_maptools.html) was applied to draw the heatmap and sort out the TMB value of each sample of high- and low-risk groups.

The failure of DDR may be an inducing factor of tumorigenesis [33]. Meanwhile, the DDR caused by radiotherapy and chemotherapy can also lead to the chemoradiotherapy tolerance of tumor cells [34]. Purposefully uniting inhibitor of DDR-related gene [35] and chemotherapy drugs [36] has gradually become a novel topic in tumor research, including ovarian cancer [37, 38]. The limma package was used to analysis the different expression of 276 genes from cardinal DDR and related pathways [39] to observe their expression pattern between high- and low-risk groups. Simultaneously, the association between risk groups and gene mutation of OV was more comprehensively surveyed by comparing the MSIsensor Score (MSIS), Fraction Genome Altered (FGA) and Aneuploidy Score (AS).

CNV was defined as the variation of DNA fragment between 1 KB and 3 MB, which is widely distributed in the human genome, and greatly enriches the diversity of genetic variation [40]. To compare CNV levels between high-risk and low-risk subtypes, masked copy number segment of TCGA-OV project was applied to seek mutated genes through Chi-square test.

Radical surgery combined with adjuvant chemotherapy is the basic method for the treatment of ovarian cancer, and therefore prediction of chemosensitivity of patients will help to further optimize the clinical efficacy [41]. GSE30161 [42] contained chemotherapy information of patients, and the difference of risk score between chemotherapy complete response (CR) and partial response patient (PR) was estimated via unpaired student’s t-test.

### Evaluation of immune cell infiltration

CIBERSORT algorithm (version 1.03; http://cibersort.stanford.edu/) is a machine learning method based on linear support vector regression (SVR) and highly robust to noise [43]. This algorithm is superior to other methods in terms of noise, unknown mixture content and closely related to cell types. Thus, in our study, CIBESORT algorithm was used to predict the proportion of different immune cells in TCGA-OV samples and to compare the difference between the high- and low-risk groups.

### Gene set variation analysis (GSVA) and connectivity map (CMap)

GSVA (version 1.38.0; http://www.bioconductor.org/packages/release/bioc/html/GSVA.html) is a nonparametric and unsupervised method to evaluate the enrichment of gene sets by transforming the differentially expressed gene expression level into the change of pathway level [44], and GSVA algorithm was used to assess potential changes in biological functions between different risk groups.

The CMap database is composed of drug-specific genomic expression profiles, including data of 1309 human cell lines that small biologically active molecules treated with [45]. We transformed the gene symbol of differentially expressed genes between high- and low-risk groups into the corresponding probe via GPL96 platform as the input, and negatively related small molecular compounds or drugs were gained utilizing comparison with gene expression profiles of reference in CMap.

### Statistical analysis

The Shapiro-Wilk normality test was used to measure the normality of the variables for comparisons of two groups. The statistical significance of discrepancy between normally distributed variables was calculated via unpaired Student’s t-test and the association was estimated by Pearson’s correlation coefficient. Survival rates were measured by the Kaplan-Meier method, and the significance of disparity between survival curves judged via the log-rank test. Survival predictive accuracy of prognostic models was assessed based on a time-dependent receiver operating characteristic curve (ROC) analysis. Chi-square test was applied to evaluate the difference of CNV level between high and low-risk groups in the genome. All statistical analyses were performed via R software (version 3.6.2) and two-tailed *P* < 0.05 was set at statistical significance.

## Results

### Determination of trait genes of immune infiltration for OV

**Fig. 1:**
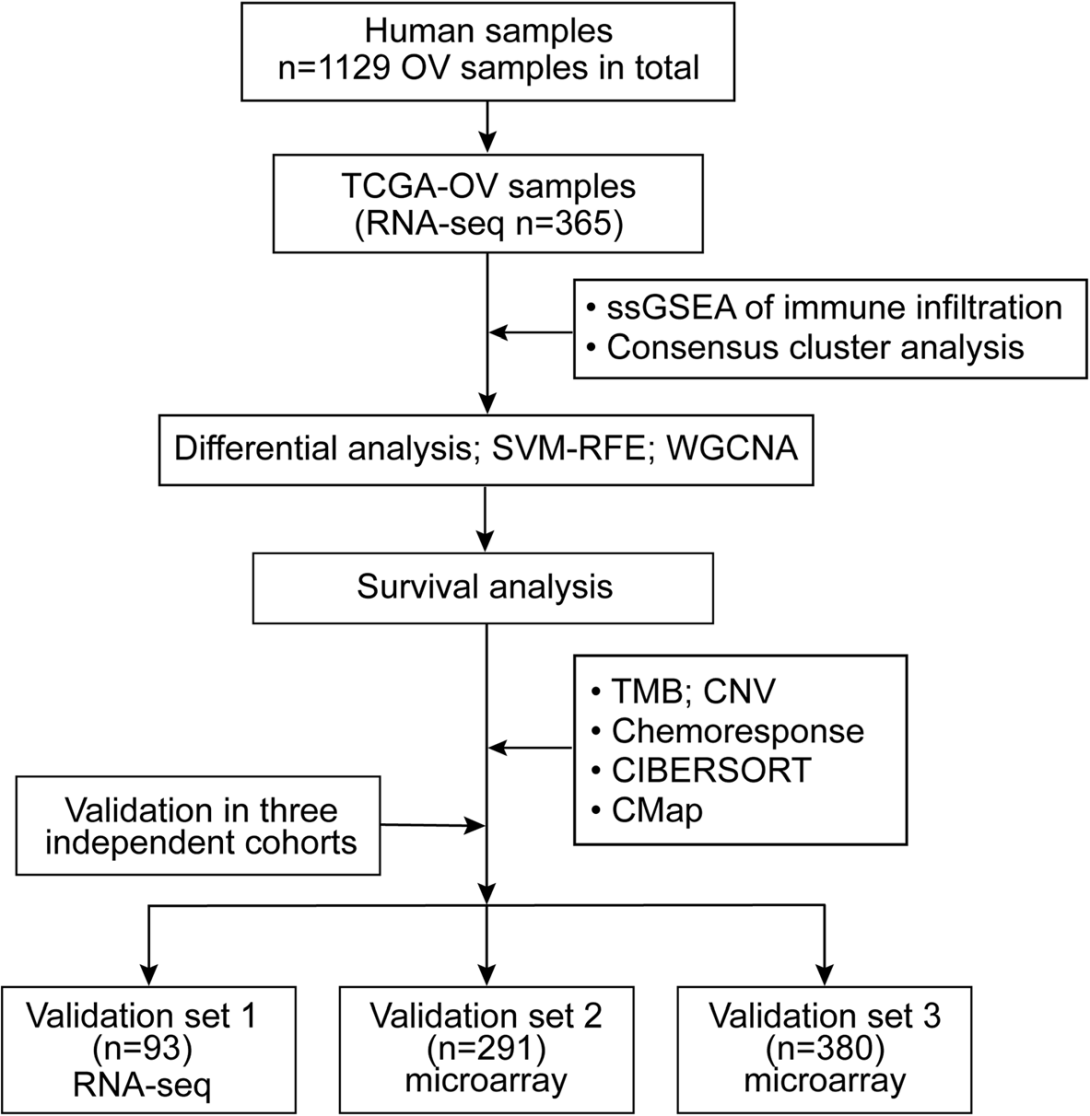
Flow chart of the study

**Fig. 2:**
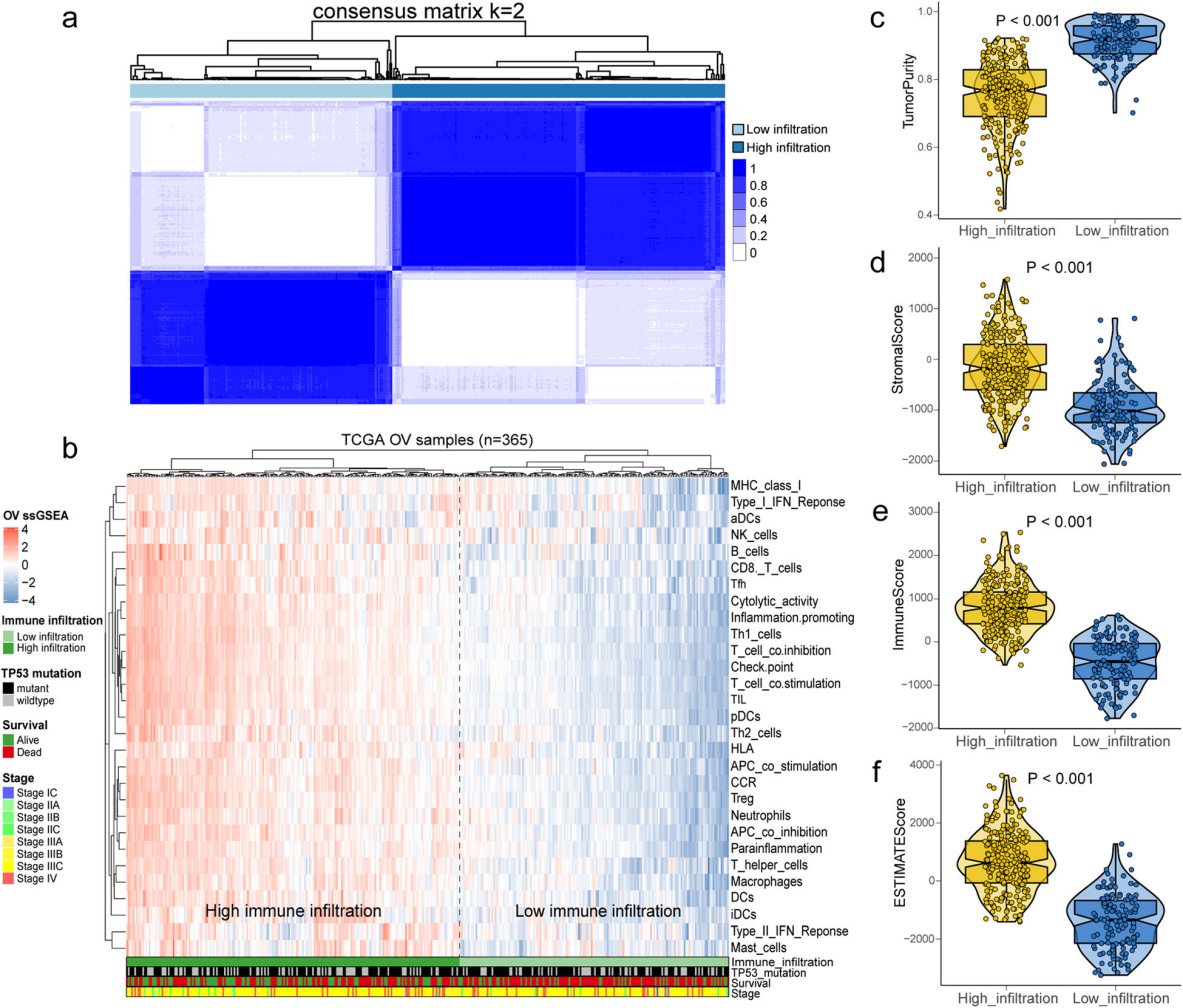
Identification of two immune infiltration subtypes in ovarian cancer (OV) cohort from The Cancer Genome Atlas (TCGA). **a** The consensus score matrix of all samples when k = 2. **b** Comparison immune profile of high and low immune infiltration groups for TCGA-OV cohort. **c-f** The distribution of tumor purity, stromal score, immune score and ESTIMATE score in high and low immune infiltration groups. **a** was generated by Consensusclusterplus (version 1.54.0); **b** was generated by ComplexHeatmap (version 2.6.2); **c**-**f** were generated by ggplot2 (version 3.2.1)

**Fig. 3:**
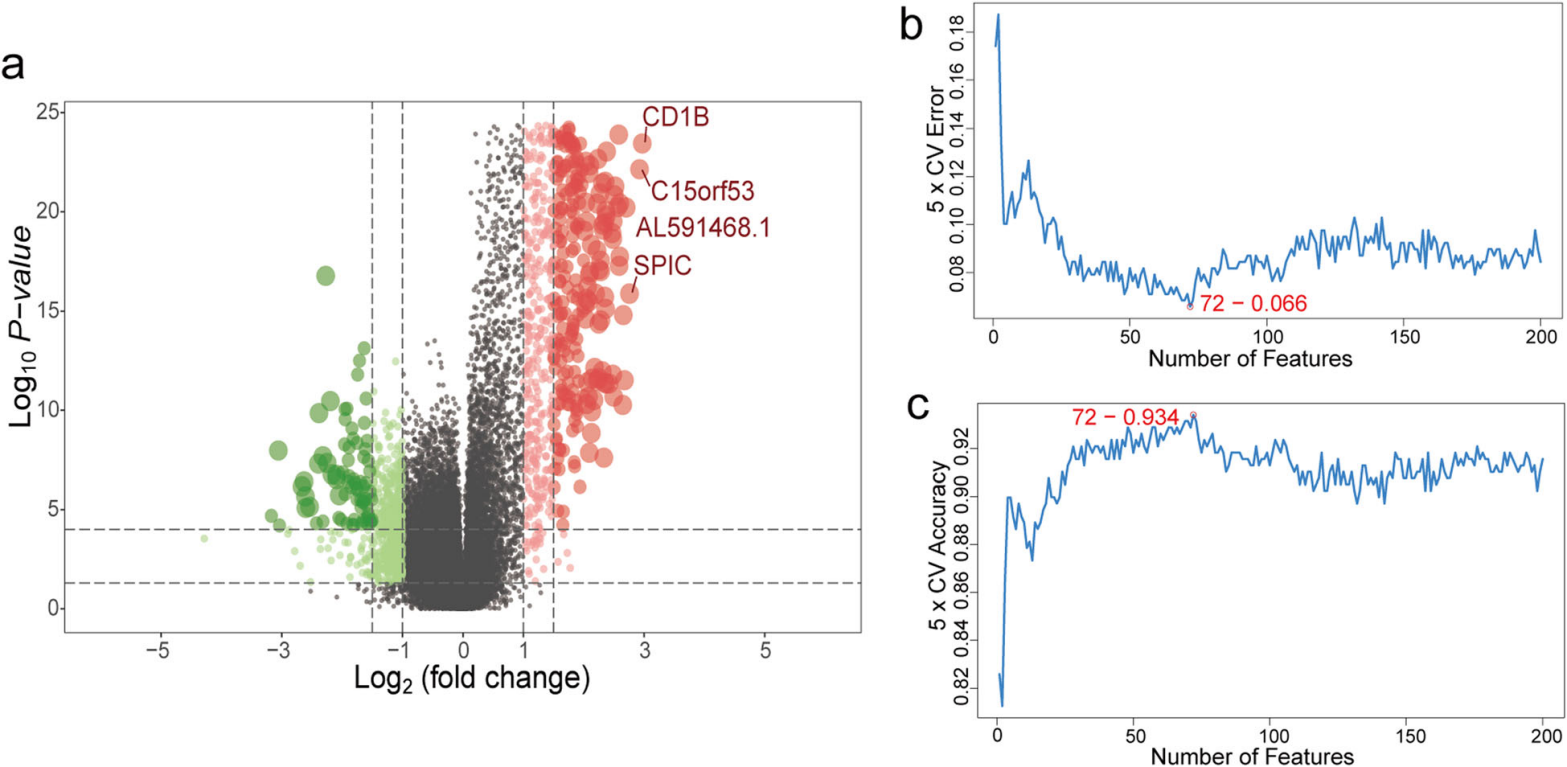
Differentially expressed genes and feature selection of genes between high and low immune infiltration groups. **a** Volcano plot of differentially expressed genes: the red dots represent significantly up-regulated genes and green dots represent significantly down-regulated genes between high and low immune infiltration groups. **b**, **c** The point highlighted indicates the lowest error rate, the highest accuracy rate and the 72 corresponding genes at both points are the best signature selected by support vector machine-recursive feature elimination (SVM-RFE) algorithm. **a** was generated by ggplot2 (version 3.2.1); **b**-**c** were generated by E1071 package (version 1.7–4)

A total of 1129 OV samples obtained from training set (*n* = 365), validation set 1 (*n* = 93), validation set 2 (*n* = 291) and validation set 3 (*n* = 380) were extracted for further analysis (Table 1). The workflow is showed in Fig. 1. Based on the ssGSEA scores of 29 gene sets, the heatmap of unsupervised cluster analysis clearly revealed two opposite clusters: high and low immune infiltration groups (Fig. 2a). Meanwhile, the analysis of ESTIMATE package showed that there were significant differences in immune activity, stromal cell score and tumour purity score between two groups (Fig. 2b). High immune group had higher immune activity and stromal cell score, while low immune group showed higher tumor purity (Fig. 2c-f). There are 1398 differentially expressed genes obtained via limma package between high and low immune infiltration groups (Fig. 3a). In a feature set consisted of 200 genes that engender the greatest effect on classification potency, a list contains 72 features was determined as the optimal subsets by SVM method, and the classification accuracy reaches 0.934 (Fig. 3b, c).

**Table 1:** Summary of the four datasets contained in the study

Dataset	Platform	Sample size	Included cohorts
Training set	Illumina	365	TCGA-OV
Validation set 1	Illumina	93	ICGC-OV-AU
Validation set 2	Affymetrix Human Genome U133A Array	291	GSE14764, GSE23554, GSE26712
Validation set 3	Illumina HumanHT-12 WG-DASL V4.0 R2 expression beadchip	380	GSE140082

### Identification of the survival-related module

Based on the clinical information of patients of TCGA-OV, the correlation analysis of module characters was carried out to find the modules significantly related to clinical features (Additional file 1: Figure S1 a-c). Three modules (green-yellow, pink, and blue) were found correlated with the prognosis of OV and relevance between GS and MM were analyzed displaying by scatter diagram (Additional file 1: Figure S1 d-f). Lastly, the union of 501 genes, containing optimal subsets (72 genes) of SVM-RFE method and 429 genes of three modules intersected with different genes expression, were included in the next procedure.

### Construction and verification of prognostic classifier

**Fig. 4:**
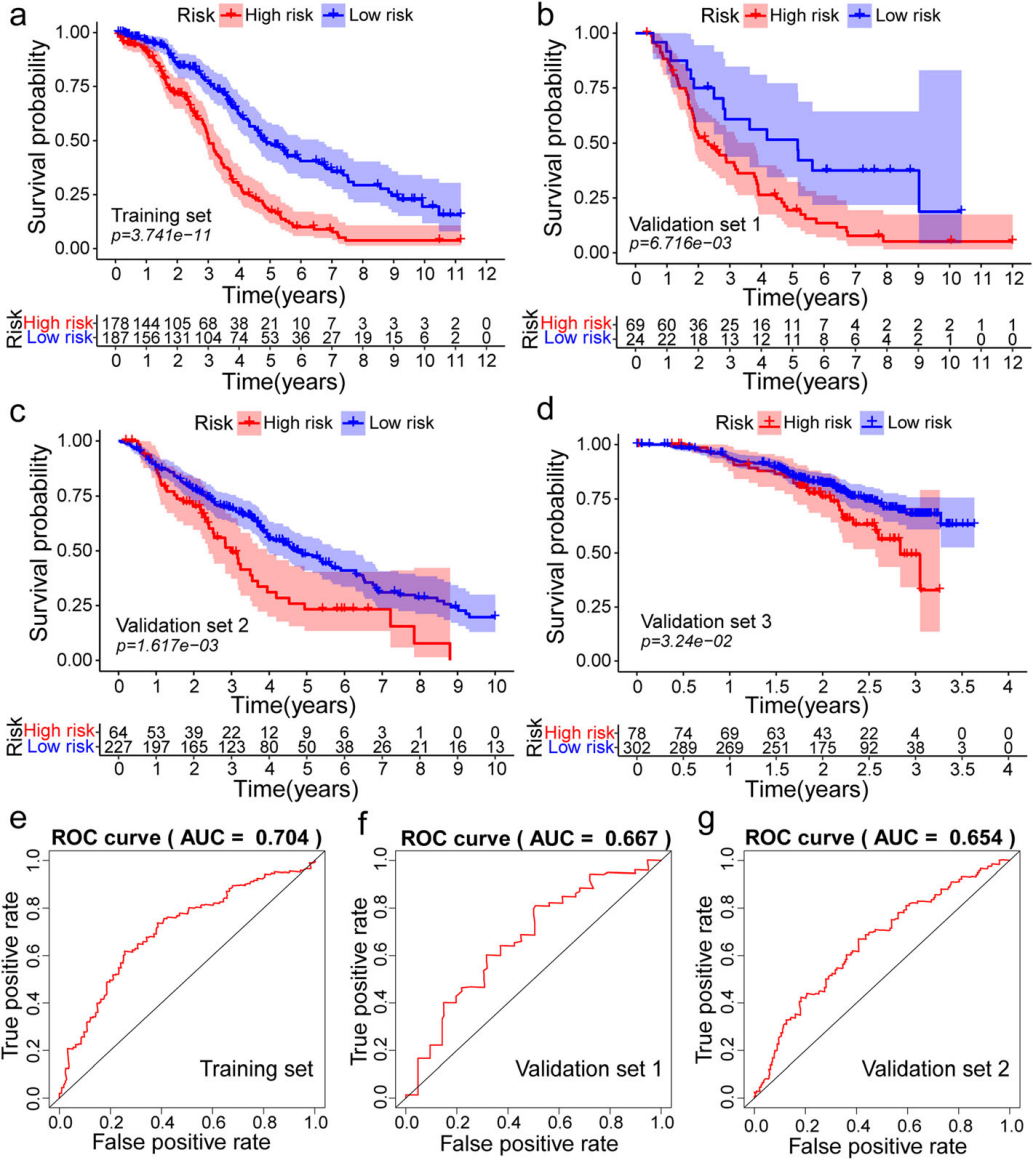
The distribution of Kaplan-Meier survival curves for overall survival (OS) in the training and validation set. **a** Kaplan-Meier survival curves for OS in the training set. **b**-**d** Kaplan-Meier survival curves for OS in the validation set. **a**-**d** were generated by survival package (version 2.41-3).

**Fig. 5:**
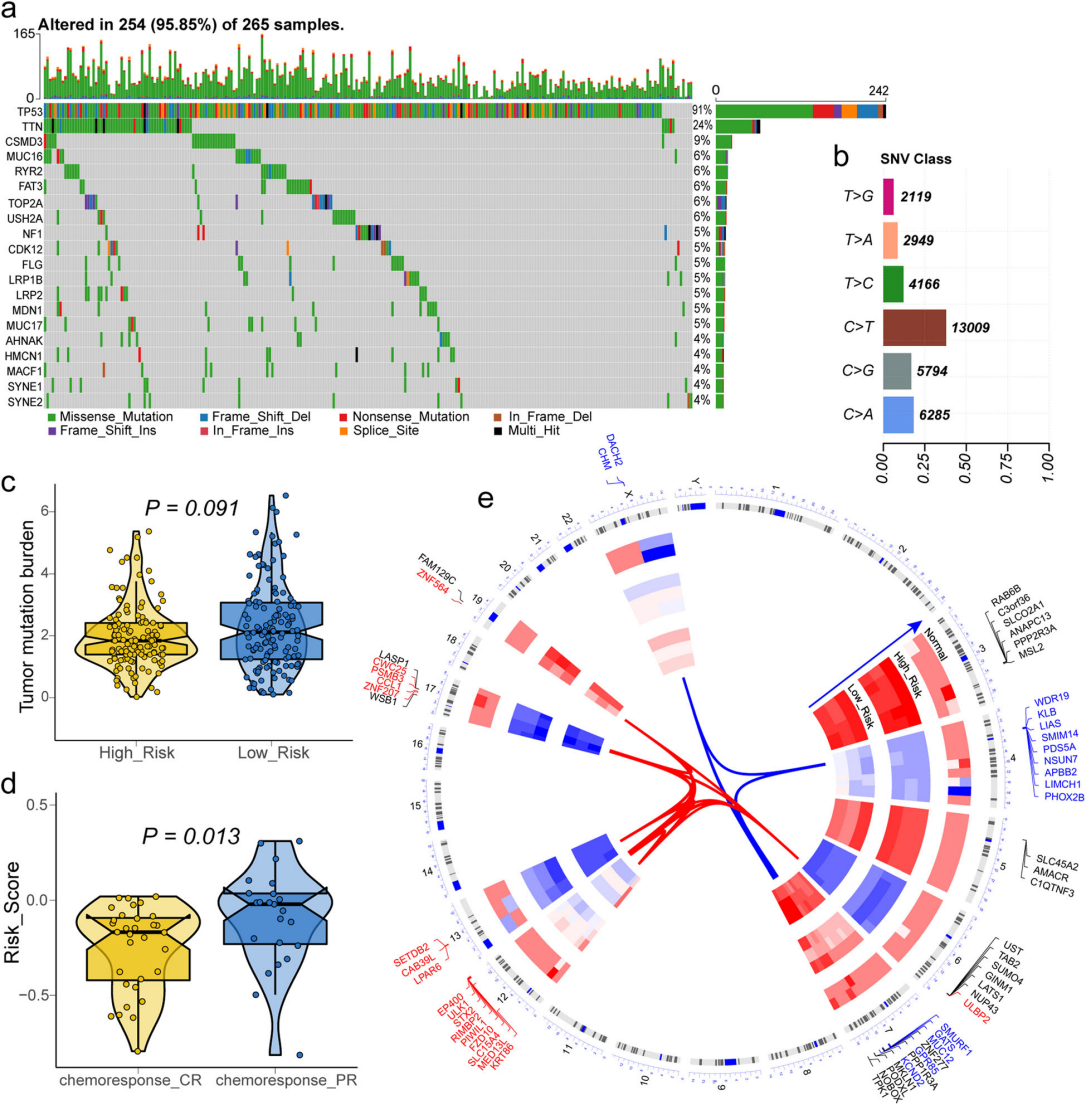
The distribution of time-dependent receiver operator characteristic (ROC) curves for overall survival in the training and validation set. **a** ROC curve of training set with area under the curve (AUC) at 5 year. **c** ROC curve of validation set with AUC at 5 year. **a**-**c** were generated by survivalROC (version 1.0.3)

Combined with the clinical information of TCGA-OV, 87 mRNA related to prognosis of OV were preliminarily screened out by univariate COX regression analysis, and top 30 mRNA sorted by *P*-value were displayed using forest plots (Additional file 2: Figure S2 a). Also, 14 key mRNA were determined significantly with prognosis by LASSO regression analysis (Additional file 2: Figure S2 c, d). Ultimately, multivariate COX regression analysis was performed to establish a prediction model with CXCL11, S1PR4, TNFRSF17, FPR1 and DHRS95 as the signature (Additional file 3: Figure S3). The formula of risk score was shown as following: risk score = 0.22135 × CXCL11 + 0.179351 × S1PR4 + 0.141478 × TNFRSF17 + 0.515099 × DHRS95. The cut-off value was identified as − 0.15 by the surv_cutpoint function in the survminer R package, and patients were divided into high-risk and low-risk groups. In TCGA training set, KM survival curve showed that the low-risk group had significantly better survival than high-risk group (*P* < 0.01, Fig. 4a). ROC curve demonstrated that the model had a great prediction ability for the prognosis of ovarian cancer patients (AUC 5-year = 0.704, Fig. 5a). Besides, in order to verify the stability of our model, the same risk scoring formula and cut off value were applied to calculate the survival of patients with ovarian cancer from ICGC and GEO database. The KM curve (*P* < 0.01, Fig. 4b, c) and 5-year survival ROC curve (Fig. 5b, c) from the validation set 1 and validation set 2 confirmed the reliability of our risk prognostic models. In GSE140082 (validation set 3), since the follow-up time of all patients were less than 5 years, we have merely conducted KM survival analysis and the results of log-rank test (*P* < 0.05) to support the efficiency of our model (Fig. 4d). To develop a quantitative method associated with clinical for predicting the survival rate of patient, a nomogram was constructed to integrate both risk score and clinical features (Additional file 4: Figure S4).

### The difference of TMB, DDR and CNV levels between high- and low-risk groups

**Fig. 6:**
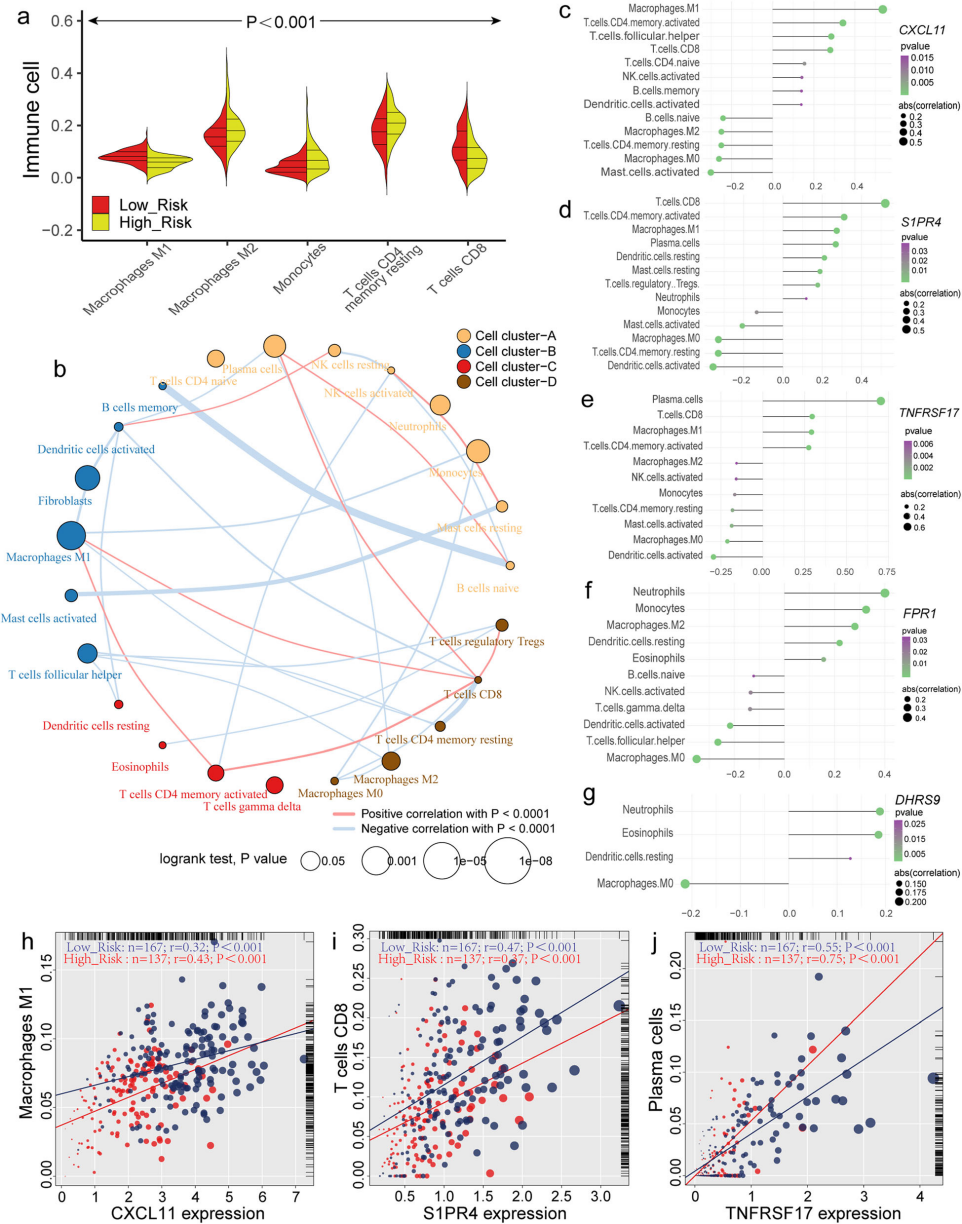
**a** Landscape of mutation profile in TCGA-OV samples. Mutation information of each gene in each sample was shown in the waterfall plot, in which various colors with annotations at the bottom represented the different mutation types. The barplot above the legend exhibited the mutation burden. **b** Summary of single nucleotide variants (SNV) with statistical calculations. **c** Tumor mutation burden (TMB) level in high-risk and low-risk groups. **d** The difference of risk score of TCGA-OV patient with complete and partial response for chemotherapy. **a**-**b** were generated by Maptools (version 1.0-2); **c**-**d** were generated by ggplot2 (version 3.2.1)

**Fig. 7:**
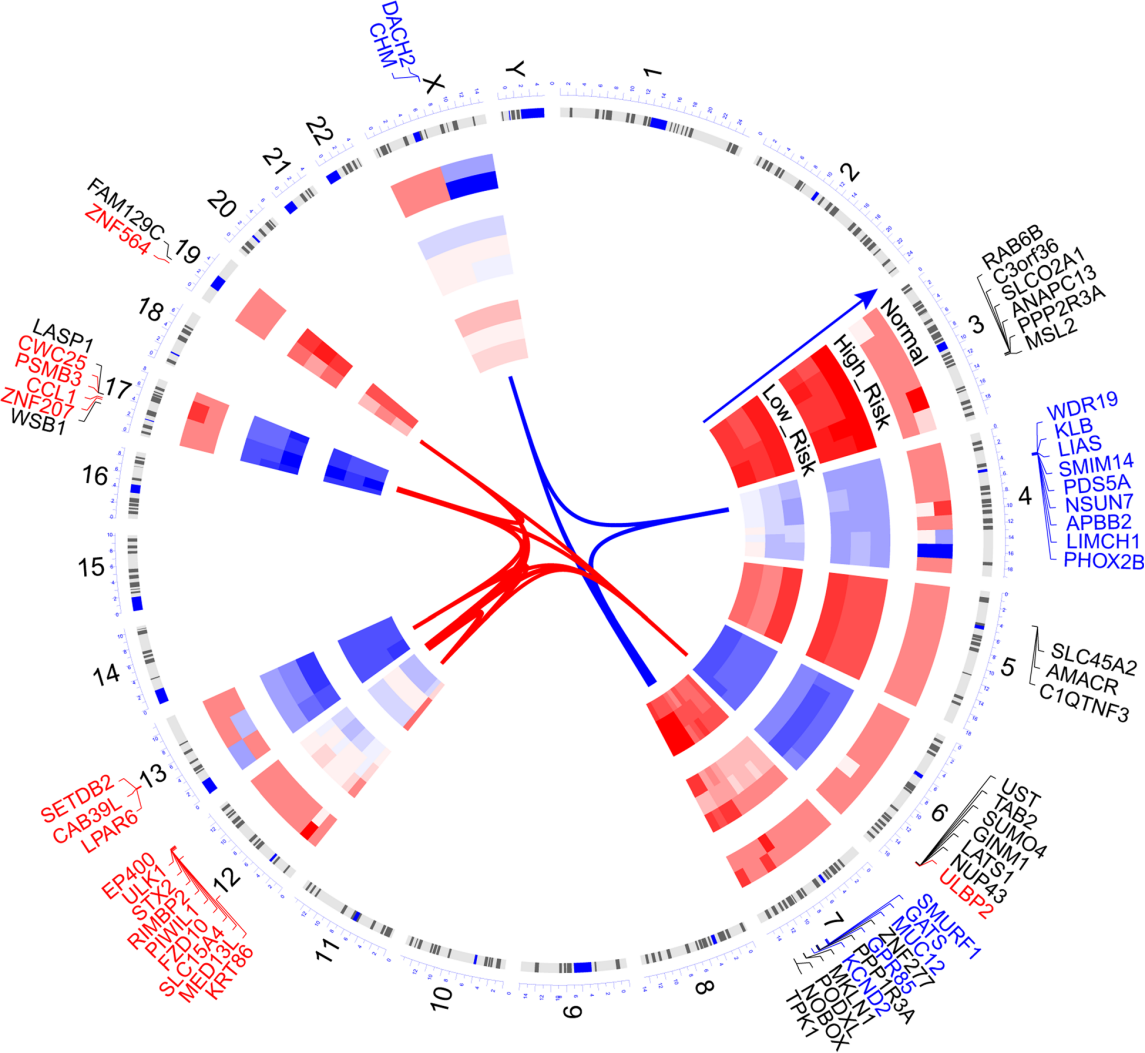
Circus plots shows the difference of copy number variations (CNV) level among low-risk, high-risk patient of TCGA-OV and normal group. The graph reflects location of variant genes on chromosome, Red genes represent exerting amplification of copy number (> 0.1) while blue genes mean deletion (< -0.1), and black genes reflect -0.1~0.1 CNV level between high- and low-risk group. Figure was generated by OmicCircos (version 1.28.0)

In the cohort of OV patients bring into the construction of survival model, 265 samples were detected for gene mutation (Fig. 6a). In brief, these mutations are sorted according to different classification categories, among which missense mutations account for the majority, and C > T is the most frequent single nucleotide variation (SNV) in ovarian cancer (Fig. 6b). In addition, we compared the TMB between the high- and low-risk groups. Although no significant difference was found between the two groups (*P* = 0.091, Fig. 6c), further study based on large samples to examine the association between the progression of OV and TMB is still needed. Through the analysis of limma package, it was found that DDR-related genes had no significantly different expression between high- and low-risk groups, and the range of logFC was − 0.25-0.14. Heatmap was conducted to show the expression of genes with the top quarter variance of TCGA-OV cohort (Additional file 5: Figure S5 a). Meanwhile, there were no significantly different distribution of three OV mutation-related parameters (MSIS, FGA and AS) between high- and low-risk groups (Additional file 5: Figure S5 b-d). By chi-square test, it was found that the location of genome CNV level difference between high- and low-risk groups. Among them, copy number deletion mainly occurs on the fourth chromosome, while copy number duplication was investigated on the 12 chromosomes (Fig. 7). The risk score of 55 patients recorded in response to chemotherapy was summarized, and it was observed that the risk score of CR patient was significantly lower than PR patient (Fig. 6d).

### The difference of fraction of immune cells between high and low-risk groups

**Fig. 8:**
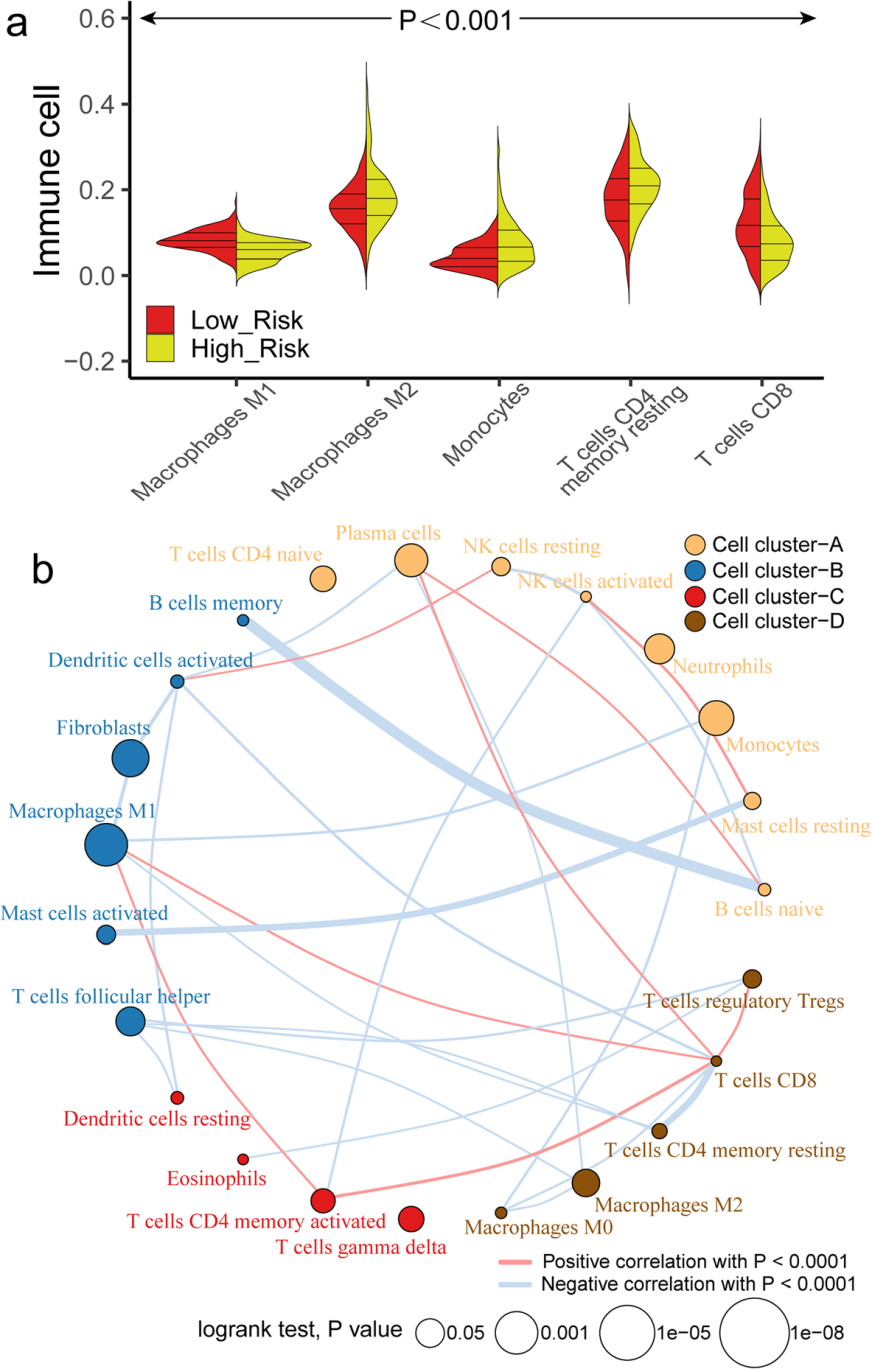
The landscape of immune infiltration in the TCGA cohort. **a** The Violin plot shows the significant difference (*P* < 0.001) of immune cell fractions between high-risk and low-risk subgroup. **b** The interaction between 22 immune cells in TCGA-OV samples. The size of circle indicated the effect of each immune cell on the prognosis, and *P* value was operated by Log-rank test. **a** was generated by ggplot2 (version 3.2.1); **b** was generated by Igraph (version 1.2.4.2)

**Fig. 9:**
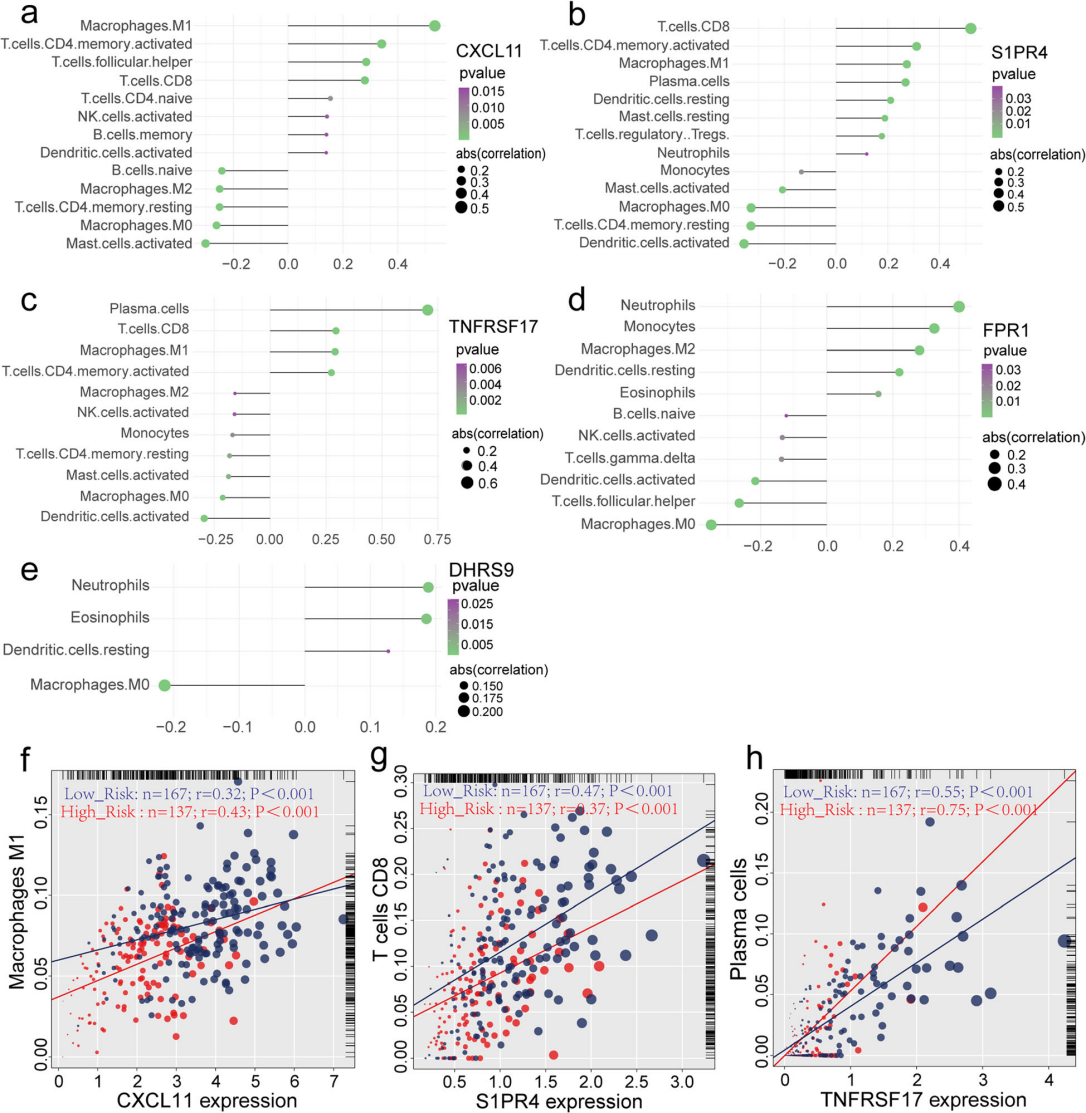
**a-e** Correlation between expression of 5 genes bring into classifier (CXCL11, S1PR4, TNFRSF 17, FPR1 and DHRS95) and immune cells in samples from TCGA-OV cohort (*P* < 0.05). **f-h** Dot plot of Pearson correlation analysis reflecting the relevance betweenthe expression of CXCL11 and Macrophages M1, S1PR4 and T cells CD8, TNFRSF17 and Plasma cells. **a-e** were generated by Ggstatsplot (version 0.6.5); **f-h** were generated by R base graphics

The distribution of immune cells in 365 OV samples was evaluated by CIBERSORT package. There are significant differences in the composition of immune cells in OV patients with different risk groups (Fig. 8a). With the increase of risk score, the proportion of Monocyte cells and M2 macrophages increases gradually with the decrease of CD8 T cells and M1 macrophages. Cluster analysis showed that the proportion of immune cells in TCGA-OV patients was divided into four categories (Fig. 8b). Simultaneously, fraction of M1 macrophages, T cells CD8, plasma cells were highly correlated with the expression of mark genes (Fig. 9a-e), especially CXCL11, S1PR4 and TNFRSF17 (Fig. 9f-h).

### Biological mechanism and potential small molecule drugs for deteriorating ovarian cancer

GSVA was used to analyze the difference between the two risk groups in biological phenotypes. There was a significant difference in the level of interferon (IFN) response between the two groups, which may be an important factor lead to poor prognosis in the high-risk group (Additional file 6: Figure S6). To further find out the effective drug molecules for the advanced treatment of ovarian cancer, the different genes expression between high- and low-risk groups were put into the quick query of CMap website and matching drug pathway molecules with the highest degree of compliance were found. According to the score size and *P*-value, we sort the top 50 comprehensive descending list of the drug molecules with the most significant negative correlation (Additional file 7: Figure S7). Notably, adrenergic receptor antagonists and cyclooxygenase inhibitors are the most frequent drug mechanisms.

## Discussion

The prognosis of patients with OV is poor and have great individual differences [46–48], so it is necessary to understand the complicated pathogenesis of OV from the perspective of genomics, to explore the molecular mechanism of prognosis differences among OV subtypes. Tumour microenvironment not only creates various favorable conditions for tumour growth, invasion and metastasis [49–51], but also plays a vital role in inducing drug resistance of tumour cells [52, 53]. Therefore, it is vital to investigate the microenvironment of OV and the influence of related factors on the biological process of OV. Meanwhile, many evidences show that biomarkers, especially genes, help boost diagnosis and treatment of cancer [54, 55]. In this study, we used ssGSEA to estimate the enrichment degree of 29 gene sets in each sample of TCGA-OV, including infiltration degree of immune cells and activity of immune-related function, and the most valuable characteristic variables of immune infiltration were worked out utilizing SVM-RFE. Then LASSO-Cox analysis was conducted to identify the five most valuable genes related to immune infiltration that significantly influenced prognosis. The stability and efficiency of the prediction model are verified in independent datasets from ICGC and GEO. The survival model established in this study spans multiple platforms. Although the reliability of the model is inferior to clinical model research based on single batch and large sample, our research method also has some advantages. According to the analysis of the experimental data conducted by Eukirchen GM [56], it found that the sensitivity of the data information obtained by microarray technology is high while the test specificity is relatively low, leading to the high true negative and false positive rate. Compared with microarray, high-throughput DNA sequencing has higher test specificity but lower test sensitivity, therefore it has higher true positive and false negative rate. So it is clear that the two technology integrated have great potential to obtain results more reliable [57]. The prediction efficiency of our model can be verified through gene expression matrixes of multiple platforms, which confirms the reliability and feasibility of our research.

Among five gene markers bring into targets of the classifier, CXCL11 is located in human chromosome 4q21.2 belonging to the ELRCXC chemokine family [58, 59], whose receptors are CXCR3 and CXCR7 [60]. Cancer cells can produce CXCL11 by autocrine or release CXCL11 via regulating tumour stromal cells in the microenvironment [61]. Dehydrogenase/family member 9 (DHRS9) is a member of the short chain dehydrogenase/reductase family [62]. Studies have shown that DHRS9 is involved in the biosynthesis of all trans-retinoic acid and exerts an anti-tumour role by inhibiting the proliferation of tumour cells, including acute promyelocytic leukemia, squamous cell carcinoma, neurocytoma and hepatocellular carcinoma [63–65]. The formyl peptide receptor 1 (FPR1), a G-protein-coupled receptor expressed by bone marrow-derived cells [66], participates in activation of immune cell induced by N-formyl peptide [67, 68]. S1PRs is the ligand of lipid second messenger S1P, exerts an important role in the physiological process of cell proliferation, differentiation, migration and immune response [69]. Among them, S1PR4 triggers the activation and polarization of immune cells, rather than the migration of immune cells, affecting the adaptive immunity [70]. The tumour necrosis factor receptor superfamily 17 (TNFRSF17) is regarded as a member of TNFRSF, preferentially expressed in mature B lymphocytes and has a positive effect on the development of B cells and autoimmune response [71]. Researches from Chae SC suggested that TNFRSF17 may be a candidate gene associated with the pathogenesis of colon cancer and haplotype of TNFRSF17 polymorphism appears to be a marker of susceptibility to colon cancer [72]. To sum up, the five gene markers are rarely discussed in the previous study of ovarian cancer and should be put more emphasis on.

TMB is an important index to reflect the somatic mutation accumulation, and it was viewed as a biomarker to select patients who benefit from immune-checkpoint treatment [73]. In the checkmate 032 clinical trial, 401 patients with end stage lung cancer were divided into three groups according to the level of TMB, and the results showed that patients with high TMB patients are superior to other patients both in treatment efficiency and median survival [74]. In our research, no significant difference was found in the level of TMB between high and low-risk patients. Therefore, more research should be focused on the screening of OV patients who might benefit from immunotherapy via TMB level. In addition, previous confirmed that aneuploidy is the dynamic of tumour development, and tumour recurrence is associated with the distribution and effect of protooncogene occurring somatic copy number variation [75, 76]. Through chi-square test, we gained a group of genes with significant CNV difference between high and low-risk groups. Furthermore, compared with low-risk group, high-risk group contained deletion of copy number on the 4th chromosome and duplication of copy number on the 12th chromosome.

Tumour-associated macrophages (TAMs) are macrophages infiltrated in tumour tissue, which mainly comes from circulating blood monocytes released from bone marrow [77]. M1 TAMs, characterized by high expression of IL-12 and low expression of IL-10, can present tumour-specific antigen and inhibit tumour development [78]. On the contrary, M2 TAMs, with high expression of IL-10 and low expression of IL-12, is a vital subtype which can promote tumor growth and chemoresistance [79]. Many studies have revealed that there is a significant correlation between the polarization of TAMs and prognosis of ovarian cancer [80, 81]. Reinartz et al. indicated that high-density CD163 + M2 TAMs was associated with advanced and poor prognosis of epithelial ovarian cancer [82]. Zhang et al. demonstrated that the density of TAMs in advanced ovarian cancer increased while the ratio of M1/M2 decreased significantly, and the survival period of TAMs patients with high M1/M2 was prolonged [83]. The primary CD8+ T cells were stimulated by the antigen-specific antigen of cancer cells presented by APC, and differentiated into cytotoxic CD8+ T cells with specific killing effect on tumour under the stimulation of multiple cytokines [84]. Eiichi Sato performed a detailed immunohistochemical evaluation of TILs in epithelial ovarian cancer and found that intraepithelial CD8 TIL was the only subtype associated with improved survival [85]. Jon Røikjaer Henriksena discovered that among all patients diagnosed with high-grade serous carcinoma (HGSC) in Denmark, the median OS was 37 and 25 months in patients with high and low-level of CD8 T cells (*P* = 0.0008). In multivariate analysis, high quantity of CD8 T cells was an independent marker for favorable OS (HR = 0.72, *P* = 0.020) [86].

Besides, GSVA analysis displays the evidence that the poor prognosis of high-risk OV patient is associated with low-level interferon response. Interferon participates in multiple life activities, such as antiviral infection, cell proliferation and immune response [87]. Also, IFN plays a crucial role in tumor immunity. On the one hand, IFN exerts an anti-tumour effect, suppresses tumour proliferation, and perform immune elimination by activating cytotoxic lymphocytes (CTLs), dendritic cells (DCS) and other immune cells [88]; On the other hand, IFN-γ upregulates PD-L1 and cause programmed death of immune cells, which enables tumour cells to escape the destructive effect of the immune system, resulting in anti-tumour treatment tolerance [89]. Therefore, it is necessary to ensure that patients can benefit from immunotherapy while assuring the stable antitumor effect of IFN.

The value of immunotherapy in OV is still in the research stage. Food and Drug Administration approved monoclonal antibodies against programmed death-1 (PD-1) for the treatment of solid tumors including OV with high microsatellite instability and mismatch repair defects [90]. Preliminary studies have shown that the current immunotherapy has limited efficacy in OV, which may be due to the heterogeneity of the tumor, the lack of antigen targets and expression of human lymphoid antigen, the high expression of immunosuppressive molecules and the low infiltration of immune cells in the ovarian tumor microenvironment [91–93]. Immune checkpoint blockade (ICB) therapy is still the most promising and most popular immunotherapy for OV [94]. However, the objective response rate of ICB is not optimistic, which may be changed by combination with other tumor therapies [95]. Further investigation to explore the molecular markers which can predict the efficacy of immunotherapy in OV to screen the appropriate immunotherapy population is needed.

Our research has presented some advantages. Firstly, the samples of OV patient comes from multiple databases, and the sample size is large enough which can better ensure the stable efficacy of the model. Secondly, the procedure of screening and analysis is clear and orderly via various machine learning methods, and finally obtain genes with feature of immune infiltration and value of survival prediction to ensure that the final screened genes for modeling have a strong biological background and can guide clinical treatment, especially the sensitivity of patients to chemotherapy. However, there are also some limitations. First of all, although we are committed to elucidate the correlation between genes for modeling and immune infiltration, there is no relevant dataset about immunotherapy of ovarian cancer to verify the efficacy of our model and to screen OV patient who may benefit from immunotherapy. More importantly, we lack our own cohort of OV patient cohort to further prove the function of our model.

## Conclusions

In conclusion, we focused on prognostic genes associated with immune infiltration, and picked five potential targets verified via 1129 OV samples which may provide some clues and landscape for clinical treatment of OV.

## Supplementary Information

**Additional file 1: Figure S1.** Identification of survival-related modules associated with the clinical information of ovarian cancer by weighted gene co-expression network analysis (WGCNA). (a) Analysis of the scale-free fit index and the mean connectivity for various soft-thresholding powers. (b) Clustering dendrogram of all differentially expressed genes, and each module represents a cluster of related genes and was assigned a unique color. (c) Heatmap of the correlations and differences in the modules associated with overall survival of ovarian cancer. (d) A scatter plot of gene significance for OS status vs. module membership in the green-yellow module. (e) A scatterplot of gene significance for Stage vs. module membership in the blue module. (f) A scatter plot of gene significance for OS status vs. module membership in the pink module. (d)-(f) showing a highly significant correlation between gene significance and module membership in modules. (a)-(f) were generated by WGCNA (version 1.69).

**Additional file 2: Figure S2.** Constructing the prognostic gene classifier by the univariate cox regression and the Lasso regression analysis. (a) The top 30 most significant prognostic genes of the training set calculated by univariate cox regression. (b), (c) Determination of the number of factors through the Least absolute shrinkage and selection operator analysis (LASSO) analysis. (a) was generated by survival package (version 2.41–3); (b)-(c) were generated by glmnet package (version 3.0–1).

**Additional file 3: Figure S3.** Forest plot illustrating the multivariate regression analysis results of each gene in five mRNA risk signature. The figure was generated by survminer package (version 0.4.3).

**Additional file 4: Figure S4.** Survival nomogram of TCGA-OV samples. An individual patient’s value is located on each variable axis, and a line is drawn upward to determine the number of points received for each variable value. The sum of these numbers is located on the Total Points axis, and a line is drawn downward to the survival axes to determine the likelihood of 5-year survival. The figure was generated by Regression Modeling Strategies (version 6.0–1).

**Additional file 5: Figure S5.** Visualization of expression of DDR-related genes and aspects of mutation status between high- and low-risk groups. (a) Heatmap showing that the expression levels of DDR-related genes were comparatively symmetrical between two subgroups. (b)-(d) The violin plots present the distribution of three features of mutation between two subgroups. (a) was generated by Pheatmap (version 1.0.12); (b)-(d) were generated by ggplot2 (version 3.2.1).

**Additional file 6: Figure S6.** Heatmap of each compound of Connectivity Map (CMap) that shares the targeted mechanism of action. The figure was generated by ComplexHeatmap (version 2.6.2).

**Additional file 7: Figure S7.** Differences in pathway activities estimated per TCGA-OV sample via gene set variation analysis (GSVA) between high-risk and low-risk subgroup. The figure was generated by ggplot2 (version 3.2.1).

## Data Availability

The datasets used and analysed during the current study are available from the corresponding author on reasonable request. The data analyzed in this study are openly available and can be found here: The Cancer Genome Atlas (TCGA, https://tcga-data.nci.nih.gov/tcga/), International Cancer Genome Consortium (ICGC, https://dcc.icgc.org/releases/current/Projects/OV-AU) and Gene Expression Omnibus database (https://www.ncbi.nlm.nih.gov/geo/query/).

## Abbreviations

OV: Ovarian cancer
TME: Tumour microenvironment
IRGs: Immune-related genes
TCGA: The Cancer Genome Atlas
ICGC: International Cancer Genome Consortium
GEO: Gene Expression Omnibus
ssGSEA: Single-sample Gene Set Enrichment Analysis
SVM-RFE: Support vector machine-recursive feature elimination
DEGs: Differential expression genes
WGCNA: Weighted gene co-expression network analysis
LASSO: Least absolute shrinkage and selection operator analysis
OS: Over survival
TMB: Tumour mutation burden
DDR: DNA damage repair
CNV: Copy number variation
GSVA: Gene set variation analysis
CMap: Connectivity map
ROC: Receiver operating characteristic curve
DHRS9: Dehydrogenase/family member 9
FPR1: The formyl peptide receptor 1
TNFRSF17: The tumour necrosis factor receptor superfamily 17
TAMs: Tumour-associated macrophages

## References

[1] Matulonis UA, Sood AK, Fallowfield L, Howitt BE, Sehouli J, Karlan BY (2016). Ovarian cancer. Nat Rev Dis Primers.

[2] Bray F, Ferlay J, Soerjomataram I, Siegel RL, Torre LA, Jemal A (2018). Global cancer statistics 2018: GLOBOCAN estimates of incidence and mortality worldwide for 36 cancers in 185 countries. CA Cancer J Clin.

[3] Siegel RL, Miller KD, Jemal A (2020). Cancer statistics, 2020. CA Cancer J Clin.

[4] Jiang Y, Wang C, Zhou S (1873). Targeting tumor microenvironment in ovarian cancer: premise and promise. Biochim Biophys Acta Rev Cancer.

[5] Nagarsheth N, Wicha MS, Zou W (2017). Chemokines in the cancer microenvironment and their relevance in cancer immunotherapy. Nat Rev Immunol.

[6] Elinav E, Nowarski R, Thaiss CA, Hu B, Jin C, Flavell RA (2013). Inflammation-induced cancer: crosstalk between tumours, immune cells and microorganisms. Nat Rev Cancer.

[7] Goode EL, Block MS, Kalli KR, Vierkant RA, Chen W, Ovarian Tumor Tissue Analysis C (2017). Dose-response association of CD8+ tumor-infiltrating lymphocytes and survival time in high-grade serous ovarian cancer. JAMA Oncol.

[8] Odunsi K (2017). Immunotherapy in ovarian cancer. Ann Oncol.

[9] Goodell V, Salazar LG, Urban N, Drescher CW, Gray H, Swensen RE (2006). Antibody immunity to the p53 oncogenic protein is a prognostic indicator in ovarian cancer. J Clin Oncol.

[10] Kandalaft LE, Powell DJ, Singh N, Coukos G (2011). Immunotherapy for ovarian cancer: what's next?. J Clin Oncol.

[11] Topalian SL, Drake CG, Pardoll DM (2015). Immune checkpoint blockade: a common denominator approach to cancer therapy. Cancer Cell.

[12] Farkkila A, Gulhan DC, Casado J, Jacobson CA, Nguyen H, Kochupurakkal B (2020). Immunogenomic profiling determines responses to combined PARP and PD-1 inhibition in ovarian cancer. Nat Commun.

[13] Borella F, Ghisoni E, Giannone G, Cosma S, Benedetto C, Valabrega G (2020). Immune checkpoint inhibitors in epithelial ovarian cancer: an overview on efficacy and future perspectives. Diagnostics (Basel).

[14] Alcaraz-Sanabria A, Baliu-Pique M, Saiz-Ladera C, Rojas K, Manzano A, Marquina G (2019). Genomic signatures of immune activation predict outcome in advanced stages of ovarian Cancer and basal-like breast tumors. Front Oncol.

[15] Liu Y, Jing R, Xu J, Liu K, Xue J, Wen Z (2015). Comparative analysis of oncogenes identified by microarray and RNA-sequencing as biomarkers for clinical prognosis. Biomark Med.

[16] Servant N, Romejon J, Gestraud P, La Rosa P, Lucotte G, Lair S (2014). Bioinformatics for precision medicine in oncology: principles and application to the SHIVA clinical trial. Front Genet.

[17] Yin L, Cai Z, Zhu B, Xu C (2018). Identification of key pathways and genes in the dynamic progression of HCC based on WGCNA. Genes (Basel).

[18] Li J, Liu C, Chen Y, Gao C, Wang M, Ma X (2019). Tumor characterization in breast Cancer identifies immune-relevant gene signatures associated with prognosis. Front Genet.

[19] Shen S, Wang G, Zhang R, Zhao Y, Yu H, Wei Y (2019). Development and validation of an immune gene-set based prognostic signature in ovarian cancer. EBioMedicine..

[20] Vathipadiekal V, Wang V, Wei W, Waldron L, Drapkin R, Gillette M (2015). Creation of a human Secretome: a novel composite library of human secreted proteins: validation using ovarian Cancer gene expression data and a virtual Secretome Array. Clin Cancer Res.

[21] Marchion DC, Cottrill HM, Xiong Y, Chen N, Bicaku E, Fulp WJ (2011). BAD phosphorylation determines ovarian cancer chemosensitivity and patient survival. Clin Cancer Res.

[22] Denkert C, Budczies J, Darb-Esfahani S, Gyorffy B, Sehouli J, Konsgen D (2009). A prognostic gene expression index in ovarian cancer - validation across different independent data sets. J Pathol.

[23] Leek JT, Johnson WE, Parker HS, Jaffe AE, Storey JD (2012). The sva package for removing batch effects and other unwanted variation in high-throughput experiments. Bioinformatics..

[24] Foroutan M, Bhuva DD, Lyu R, Horan K, Cursons J, Davis MJ (2018). Single sample scoring of molecular phenotypes. BMC Bioinformatics.

[25] Wilkerson MD, Hayes DN (2010). ConsensusClusterPlus: a class discovery tool with confidence assessments and item tracking. Bioinformatics..

[26] Chakraborty H, Hossain A (2018). R package to estimate intracluster correlation coefficient with confidence interval for binary data. Comput Methods Prog Biomed.

[27] Huang S, Cai N, Pacheco PP, Narrandes S, Wang Y, Xu W (2018). Applications of support vector machine (SVM) learning in Cancer genomics. Cancer Genomics Proteomics.

[28] Wang Q, Liu X (2015). Screening of feature genes in distinguishing different types of breast cancer using support vector machine. Onco Targets Ther.

[29] Langfelder P, Horvath S (2008). WGCNA: an R package for weighted correlation network analysis. BMC Bioinformatics..

[30] Allgauer M, Budczies J, Christopoulos P, Endris V, Lier A, Rempel E (2018). Implementing tumor mutational burden (TMB) analysis in routine diagnostics-a primer for molecular pathologists and clinicians. Transl Lung Cancer Res.

[31] Chan TA, Yarchoan M, Jaffee E, Swanton C, Quezada SA, Stenzinger A (2019). Development of tumor mutation burden as an immunotherapy biomarker: utility for the oncology clinic. Ann Oncol.

[32] High TMB (2018). Predicts immunotherapy benefit. Cancer Discovery.

[33] Begg AC, Stewart FA, Vens C (2011). Strategies to improve radiotherapy with targeted drugs. Nat Rev Cancer.

[34] Lord CJ, Ashworth A (2013). Mechanisms of resistance to therapies targeting BRCA-mutant cancers. Nat Med.

[35] Kummar S, Chen A, Parchment RE, Kinders RJ, Ji J, Tomaszewski JE (2012). Advances in using PARP inhibitors to treat cancer. BMC Med.

[36] Mittica G, Ghisoni E, Giannone G, Genta S, Aglietta M, Sapino A (2018). PARP inhibitors in ovarian Cancer. Recent Pat Anticancer Drug Discov.

[37] Pujade-Lauraine E, Ledermann JA, Selle F, Gebski V, Penson RT, Oza AM (2017). Olaparib tablets as maintenance therapy in patients with platinum-sensitive, relapsed ovarian cancer and a BRCA1/2 mutation (SOLO2/ENGOT-Ov21): a double-blind, randomised, placebo-controlled, phase 3 trial. Lancet Oncol.

[38] Ledermann J, Harter P, Gourley C, Friedlander M, Vergote I, Rustin G (2012). Olaparib maintenance therapy in platinum-sensitive relapsed ovarian cancer. N Engl J Med.

[39] Knijnenburg TA, Wang L, Zimmermann MT, Chambwe N, Gao GF, Cherniack AD (2018). Genomic and molecular landscape of DNA damage repair deficiency across the Cancer genome atlas. Cell Rep.

[40] Haraksingh RR, Abyzov A, Urban AE (2017). Comprehensive performance comparison of high-resolution array platforms for genome-wide copy number variation (CNV) analysis in humans. BMC Genomics.

[41] Eisenhauer EA (2017). Real-world evidence in the treatment of ovarian cancer. Ann Oncol.

[42] Ferriss JS, Kim Y, Duska L, Birrer M, Levine DA, Moskaluk C (2012). Multi-gene expression predictors of single drug responses to adjuvant chemotherapy in ovarian carcinoma: predicting platinum resistance. PLoS One.

[43] Newman AM, Liu CL, Green MR, Gentles AJ, Feng W, Xu Y (2015). Robust enumeration of cell subsets from tissue expression profiles. Nat Methods.

[44] Hanzelmann S, Castelo R, Guinney J (2013). GSVA: gene set variation analysis for microarray and RNA-seq data. BMC Bioinformatics..

[45] Liu H, Zhou Q, Wei W, Qi B, Zeng F, Bao N (2020). The potential drug for treatment in pancreatic adenocarcinoma: a bioinformatical study based on distinct drug databases. Chin Med.

[46] Narod S (2016). Can advanced-stage ovarian cancer be cured?. Nat Rev Clin Oncol.

[47] Scaletta G, Plotti F, Luvero D, Capriglione S, Montera R, Miranda A (2017). The role of novel biomarker HE4 in the diagnosis, prognosis and follow-up of ovarian cancer: a systematic review. Expert Rev Anticancer Ther.

[48] Kossai M, Leary A, Scoazec JY, Genestie C (2018). Ovarian Cancer: a heterogeneous disease. Pathobiology..

[49] Gasser S, Lim LHK, Cheung FSG (2017). The role of the tumour microenvironment in immunotherapy. Endocr Relat Cancer.

[50] Barker HE, Paget JT, Khan AA, Harrington KJ (2015). The tumour microenvironment after radiotherapy: mechanisms of resistance and recurrence. Nat Rev Cancer.

[51] Laplane L, Duluc D, Bikfalvi A, Larmonier N, Pradeu T (2019). Beyond the tumour microenvironment. Int J Cancer.

[52] Friedmann Angeli JP, Krysko DV, Conrad M (2019). Ferroptosis at the crossroads of cancer-acquired drug resistance and immune evasion. Nat Rev Cancer.

[53] Yousefzadeh Y, Hallaj S, Baghi Moornani M, Asghary A, Azizi G, Hojjat-Farsangi M (2020). Tumor associated macrophages in the molecular pathogenesis of ovarian cancer. Int Immunopharmacol.

[54] Lu Y, Yang G, Xiao Y, Zhang T, Su F, Chang R (2020). Upregulated cyclins may be novel genes for triple-negative breast cancer based on bioinformatic analysis. Breast Cancer.

[55] Shang H, Liu ZP (2020). Network-based prioritization of cancer genes by integrative ranks from multi-omics data. Comput Biol Med.

[56] Euskirchen GM, Rozowsky JS, Wei CL, Lee WH, Zhang ZD, Hartman S (2007). Mapping of transcription factor binding regions in mammalian cells by ChIP: comparison of array- and sequencing-based technologies. Genome Res.

[57] Robertson G, Hirst M, Bainbridge M, Bilenky M, Zhao Y, Zeng T (2007). Genome-wide profiles of STAT1 DNA association using chromatin immunoprecipitation and massively parallel sequencing. Nat Methods.

[58] Nazari A, Ahmadi Z, Hassanshahi G, Abbasifard M, Taghipour Z, Falahati-Pour SK (2020). Effective treatments for bladder Cancer affecting CXCL9/CXCL10/CXCL11/CXCR3 Axis: a review. Oman Med J.

[59] Chen X, Chen R, Jin R, Huang Z (2020). The role of CXCL chemokine family in the development and progression of gastric cancer. Int J Clin Exp Pathol.

[60] Puchert M, Obst J, Koch C, Zieger K, Engele J (2020). CXCL11 promotes tumor progression by the biased use of the chemokine receptors CXCR3 and CXCR7. Cytokine..

[61] Benhadjeba S, Edjekouane L, Sauve K, Carmona E, Tremblay A (2018). Feedback control of the CXCR7/CXCL11 chemokine axis by estrogen receptor alpha in ovarian cancer. Mol Oncol.

[62] Shimomura H, Sasahira T, Nakashima C, Shimomura-Kurihara M, Kirita T (2018). Downregulation of DHRS9 is associated with poor prognosis in oral squamous cell carcinoma. Pathology..

[63] Kropotova ES, Zinovieva OL, Zyryanova AF, Dybovaya VI, Prasolov VS, Beresten SF (2014). Altered expression of multiple genes involved in retinoic acid biosynthesis in human colorectal cancer. Pathol Oncol Res.

[64] Kuznetsova ES, Zinovieva OL, Oparina NY, Prokofjeva MM, Spirin PV, Favorskaya IA (2016). Abnormal expression of genes that regulate retinoid metabolism and signaling in non-small-cell lung cancer. Mol Biol (Mosk).

[65] Kim EW, De Leon A, Jiang Z, Radu RA, Martineau AR, Chan ED (2019). Vitamin A metabolism by dendritic cells triggers an antimicrobial response against *Mycobacterium tuberculosis*. mSphere.

[66] D'Amico R, Fusco R, Cordaro M, Siracusa R, Peritore AF, Gugliandolo E (2020). Modulation of NLRP3 inflammasome through formyl peptide receptor 1 (Fpr-1) pathway as a new therapeutic target in bronchiolitis obliterans syndrome. Int J Mol Sci.

[67] Minopoli M, Polo A, Ragone C, Ingangi V, Ciliberto G, Pessi A (2019). Structure-function relationship of an Urokinase receptor-derived peptide which inhibits the Formyl peptide receptor type 1 activity. Sci Rep.

[68] Cao G, Zhang Z (2018). FPR1 mediates the tumorigenicity of human cervical cancer cells. Cancer Manag Res.

[69] Jozefczuk E, Guzik TJ, Siedlinski M (2020). Significance of sphingosine-1-phosphate in cardiovascular physiology and pathology. Pharmacol Res.

[70] Olesch C, Ringel C, Brune B, Weigert A (2017). Beyond immune cell migration: the emerging role of the Sphingosine-1-phosphate receptor S1PR4 as a modulator of innate immune cell activation. Mediat Inflamm.

[71] Lee L, Bounds D, Paterson J, Herledan G, Sully K, Seestaller-Wehr LM (2016). Evaluation of B cell maturation antigen as a target for antibody drug conjugate mediated cytotoxicity in multiple myeloma. Br J Haematol.

[72] Chae SC, Yu JI, Uhm TB, Lee SY, Kang DB, Lee JK (2012). The haplotypes of TNFRSF17 polymorphisms are associated with colon cancer in a Korean population. Int J Color Dis.

[73] Shim JH, Kim HS, Cha H, Kim S, Kim TM, Anagnostou V (2020). HLA-corrected tumor mutation burden and homologous recombination deficiency for the prediction of response to PD-(L)1 blockade in advanced non-small-cell lung cancer patients. Ann Oncol.

[74] Ready NE, Ott PA, Hellmann MD, Zugazagoitia J, Hann CL, de Braud F (2020). Nivolumab Monotherapy and Nivolumab plus Ipilimumab in recurrent small cell lung Cancer: results from the CheckMate 032 randomized cohort. J Thorac Oncol.

[75] Ahmed W, Malik MFA, Saeed M, Haq F (2018). Copy number profiling of Oncotype DX genes reveals association with survival of breast cancer patients. Mol Biol Rep.

[76] Fatima A, Tariq F, Malik MFA, Qasim M, Haq F (2017). Copy number profiling of MammaPrint genes reveals association with the prognosis of breast Cancer patients. J Breast Cancer.

[77] Yeung OW, Lo CM, Ling CC, Qi X, Geng W, Li CX (2015). Alternatively activated (M2) macrophages promote tumour growth and invasiveness in hepatocellular carcinoma. J Hepatol.

[78] Mantovani A, Marchesi F, Malesci A, Laghi L, Allavena P (2017). Tumour-associated macrophages as treatment targets in oncology. Nat Rev Clin Oncol.

[79] Lee C, Jeong H, Bae Y, Shin K, Kang S, Kim H (2019). Targeting of M2-like tumor-associated macrophages with a melittin-based pro-apoptotic peptide. J Immunother Cancer.

[80] Yin M, Shen J, Yu S, Fei J, Zhu X, Zhao J (2019). Tumor-associated macrophages (TAMs): a critical activator in ovarian Cancer metastasis. Onco Targets Ther..

[81] Maccio A, Gramignano G, Cherchi MC, Tanca L, Melis L, Madeddu C (2020). Role of M1-polarized tumor-associated macrophages in the prognosis of advanced ovarian cancer patients. Sci Rep.

[82] Reinartz S, Schumann T, Finkernagel F, Wortmann A, Jansen JM, Meissner W (2014). Mixed-polarization phenotype of ascites-associated macrophages in human ovarian carcinoma: correlation of CD163 expression, cytokine levels and early relapse. Int J Cancer.

[83] Zhang M, He Y, Sun X, Li Q, Wang W, Zhao A (2014). A high M1/M2 ratio of tumor-associated macrophages is associated with extended survival in ovarian cancer patients. J Ovarian Res.

[84] Overgaard NH, Jung JW, Steptoe RJ, Wells JW (2015). CD4+/CD8+ double-positive T cells: more than just a developmental stage?. J Leukoc Biol.

[85] Sato E, Olson SH, Ahn J, Bundy B, Nishikawa H, Qian F (2005). Intraepithelial CD8+ tumor-infiltrating lymphocytes and a high CD8+/regulatory T cell ratio are associated with favorable prognosis in ovarian cancer. Proc Natl Acad Sci U S A.

[86] Henriksen JR, Donskov F, Waldstrom M, Jakobsen A, Hjortkjaer M, Petersen CB (2020). Favorable prognostic impact of natural killer cells and T cells in high-grade serous ovarian carcinoma. Acta Oncol.

[87] Negishi H, Taniguchi T, Yanai H (2018). The interferon (IFN) class of cytokines and the IFN regulatory factor (IRF) transcription factor family. Cold Spring Harb Perspect Biol.

[88] Schmid H, Dobrovolny HM (2020). An approximate solution of the interferon-dependent viral kinetics model of influenza. J Theor Biol.

[89] Yang PM, Hsieh YY, Du JL, Yen SC, Hung CF (2020). Sequential interferon beta-Cisplatin treatment enhances the surface exposure of calreticulin in cancer cells via an interferon regulatory factor 1-dependent manner. Biomolecules.

[90] Asaoka Y, Ijichi H, Koike K (2015). PD-1 blockade in tumors with mismatch-repair deficiency. N Engl J Med.

[91] Galon J, Bruni D (2019). Approaches to treat immune hot, altered and cold tumours with combination immunotherapies. Nat Rev Drug Discov.

[92] Cai DL, Jin LP (2017). Immune cell population in ovarian tumor microenvironment. J Cancer.

[93] Binnewies M, Roberts EW, Kersten K, Chan V, Fearon DF, Merad M (2018). Understanding the tumor immune microenvironment (TIME) for effective therapy. Nat Med.

[94] Gaillard SL, Secord AA, Monk B (2016). The role of immune checkpoint inhibition in the treatment of ovarian cancer. Gynecol Oncol Res Pract.

[95] Zamarin D, Jazaeri AA (2016). Leveraging immunotherapy for the treatment of gynecologic cancers in the era of precision medicine. Gynecol Oncol.

