# Correction to: Comprehensive analysis of prognostic gene signatures based on immune infiltration of ovarian cancer

**DOI:** 10.1186/s12885-020-07724-1

**Published:** 2021-01-12

**Authors:** Shibai Yan, Juntao Fang, Yongcai Chen, Yong Xie, Siyou Zhang, Xiaohui Zhu, Feng Fang

**Affiliations:** 1 Department of Medical Oncology, the First Affiliated Hospital of Guangxi Medical University, Nanning, 530021 Guangxi Zhuang Autonomous Region China; 2 Laboratory of Experimental Cardiology, Department of Cardiology, University Medical Center Utrecht, Utrecht, 3584 CX The Netherlands; 3 Department of Obstetrics and Gynecology, The First People’s Hospital of Foshan, 81 Lingnan North Avenue, Foshan, 528000 Guangdong China; 4 Department of Pharmacology, College of Pharmacy, Shenzhen Technology University, Shenzhen, 518118 Guangdong China

**Correction to: BMC Cancer 20, 1205 (2020)**

**https://doi.org/10.1186/s12885-020-07695-3**

Following publication of the original article [1], the authors reported a typesetting error in the arrangement of Figs. 2, 3, 4, 5, 6, 7, 8 and 9, as well as their corresponding captions. The correct figures and captions are given in this correction article and the original article [1] has been corrected.

**Fig. 2:**
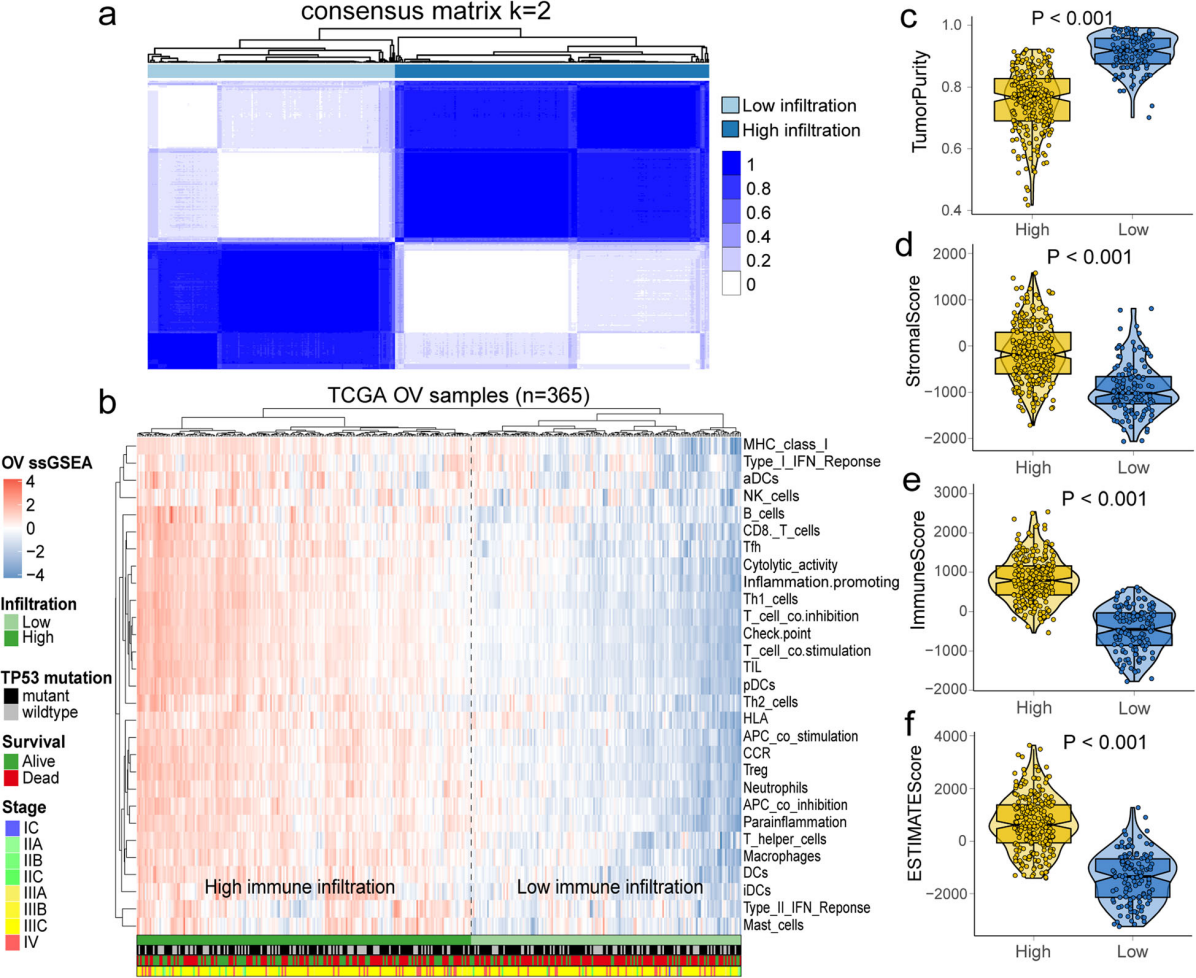
Identification of two immune infiltration subtypes in ovarian cancer (OV) cohort from The Cancer Genome Atlas (TCGA). **a** The consensus score matrix of all samples when k = 2. **b** Comparison immune profile of high and low immune infiltration groups for TCGA-OV cohort. **c**-**f** The distribution of tumor purity, stromal score, immune score and ESTIMATE score in high and low immune infiltration groups. **a** was generated by Consensusclusterplus (version 1.54.0); **b** was generated by ComplexHeatmap (version 2.6.2); **c**-**f** were generated by ggplot2 (version 3.2.1)

**Fig. 3:**
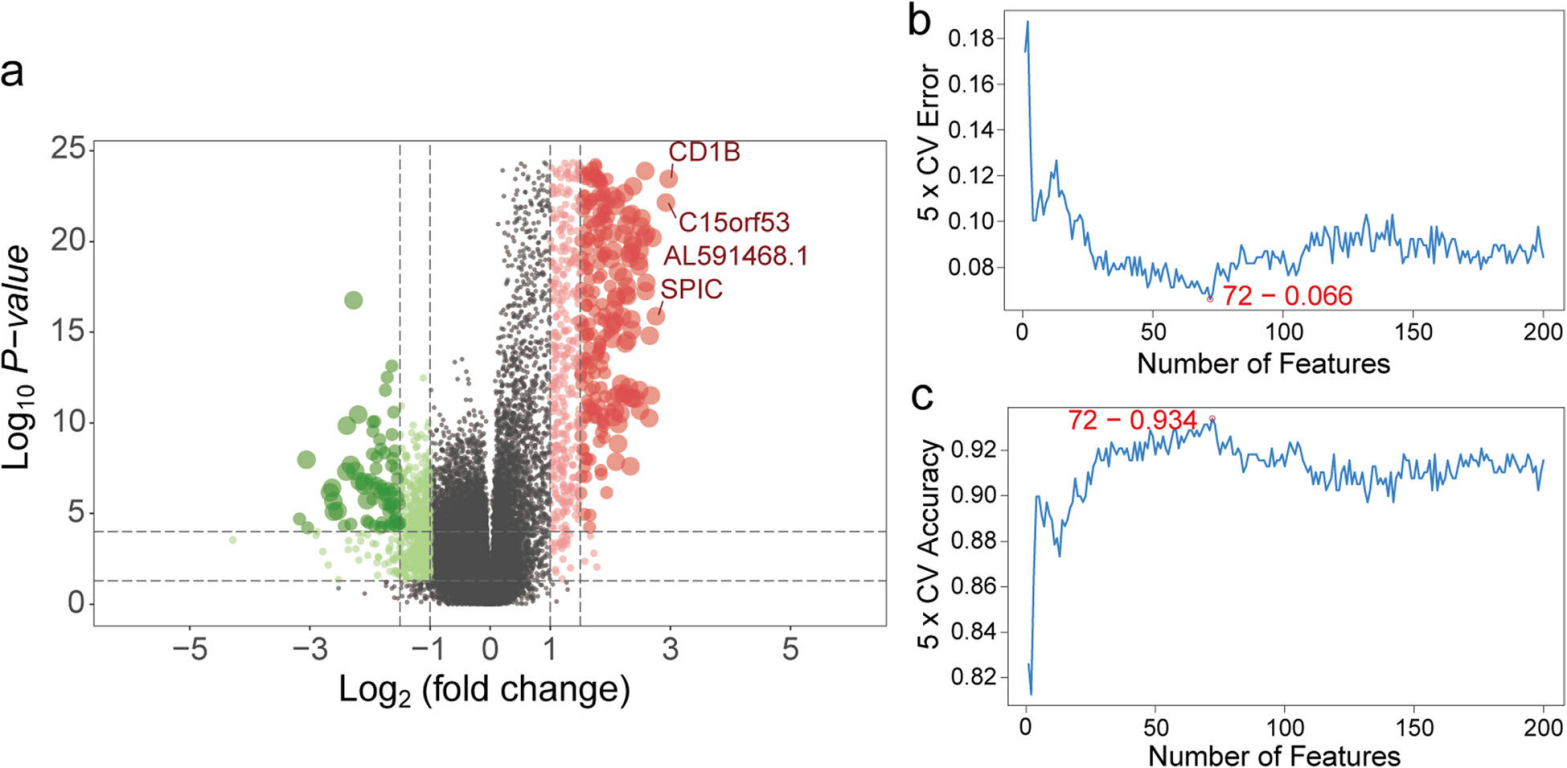
Differentially expressed genes and feature selection of genes between high and low immune infiltration groups. **a** Volcano plot of differentially expressed genes: the red dots represent significantly up-regulated genes and green dots represent significantly down-regulated genes between high and low immune infiltration groups. **b**-**c** The point highlighted indicates the lowest error rate and the highest accuracy rate; the 72 corresponding genes at both points are the best signature selected by support vector machine-recursive feature elimination (SVM-RFE) algorithm. **a** was generated by ggplot2 (version 3.2.1); **b**-**c** were generated by E1071 package (version 1.7–4)

**Fig. 4:**
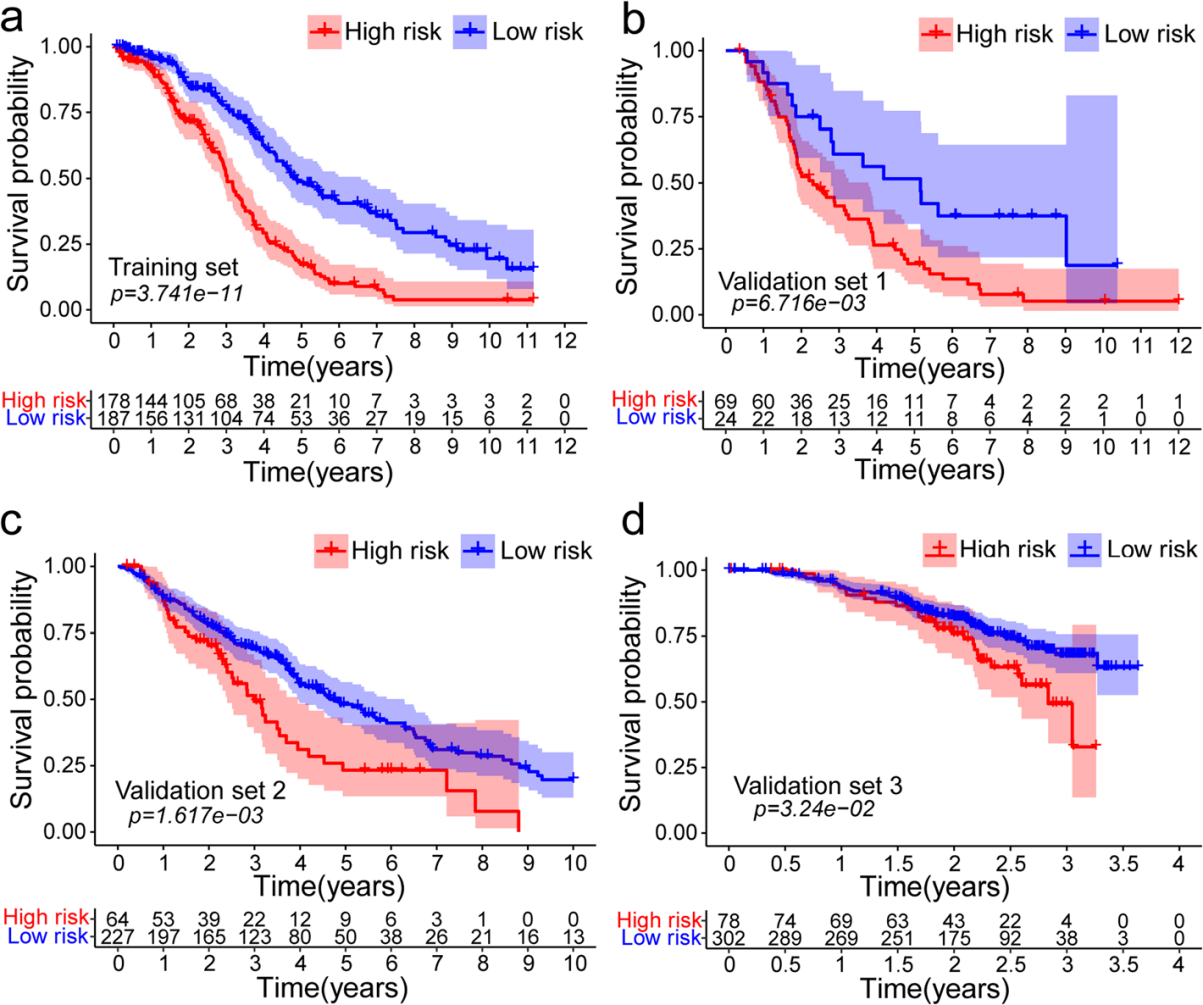
The distribution of Kaplan-Meier survival curves for overall survival (OS) in the training and validation set. **a** Kaplan-Meier survival curves for OS in the training set. **b**-**d** Kaplan-Meier survival curves for OS in the validation set. **a**-**d** were generated by survival package (version 2.41–3)

**Fig. 5:**
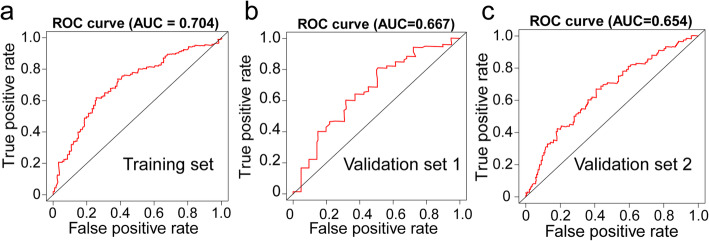
The distribution of time-dependent receiver operator characteristic (ROC) curves for overall survival in the training and validation set. **a** ROC curve of training set with area under the curve (AUC) at 5 year. **b**-**c** ROC curve of validation set with AUC at 5 year. **a**-**c** were generated by survivalROC (version 1.0.3)

**Fig. 6:**
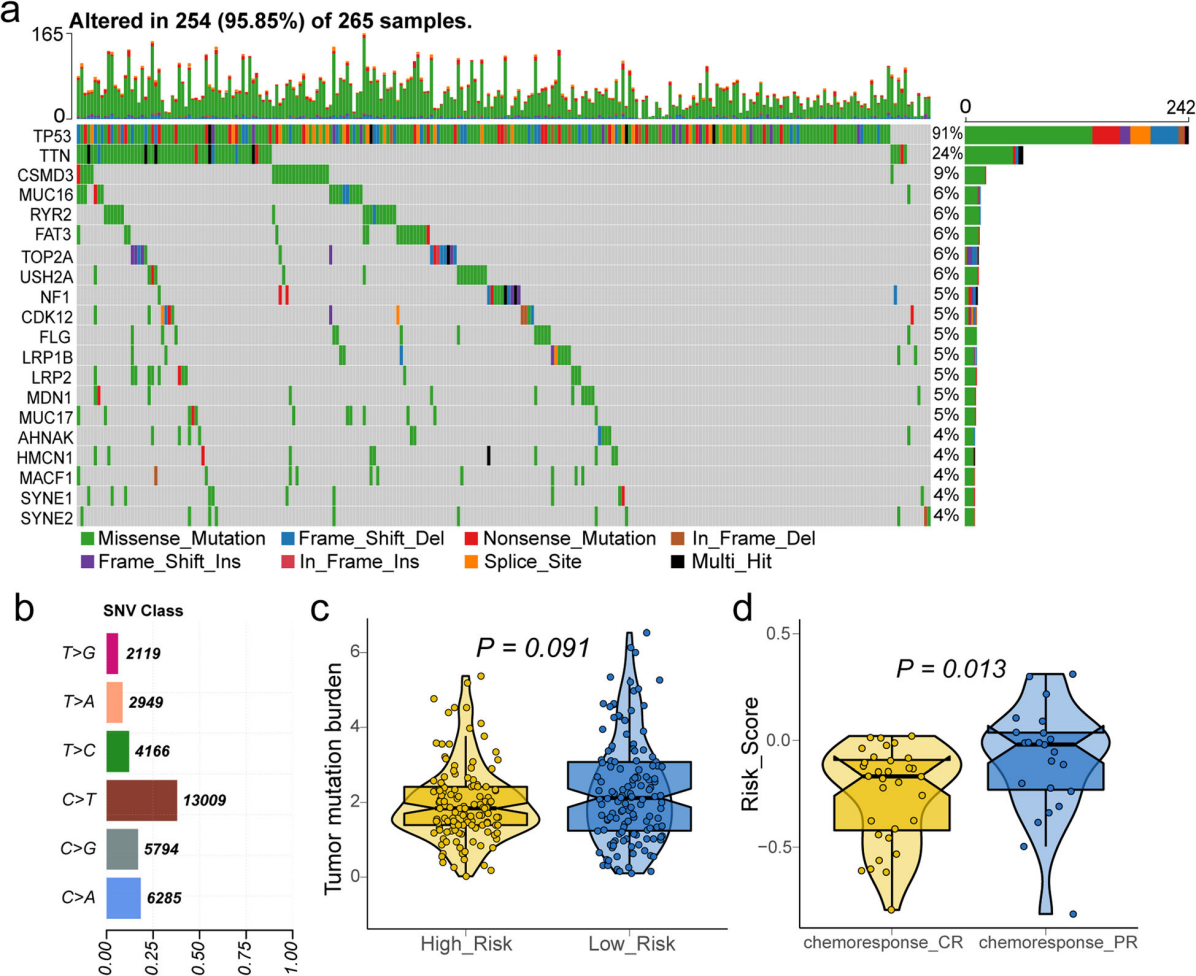
**a** Landscape of mutation profile in TCGA-OV samples. Mutation information of each gene in each sample was shown in the waterfall plot, in which various colors with annotations at the bottom represented the different mutation types. The barplot above the legend exhibited the mutation burden. **b** Summary of single nucleotide variants (SNV) with statistical calculations. **c** Tumor mutation burden (TMB) level in high-risk and low-risk groups. **d** The difference of risk score of TCGA-OV patient with complete and partial response for chemotherapy. **a**-**b** were generated by Maptools (version 1.0–2); **c**-**d** were generated by ggplot2 (version 3.2.1)

**Fig. 7:**
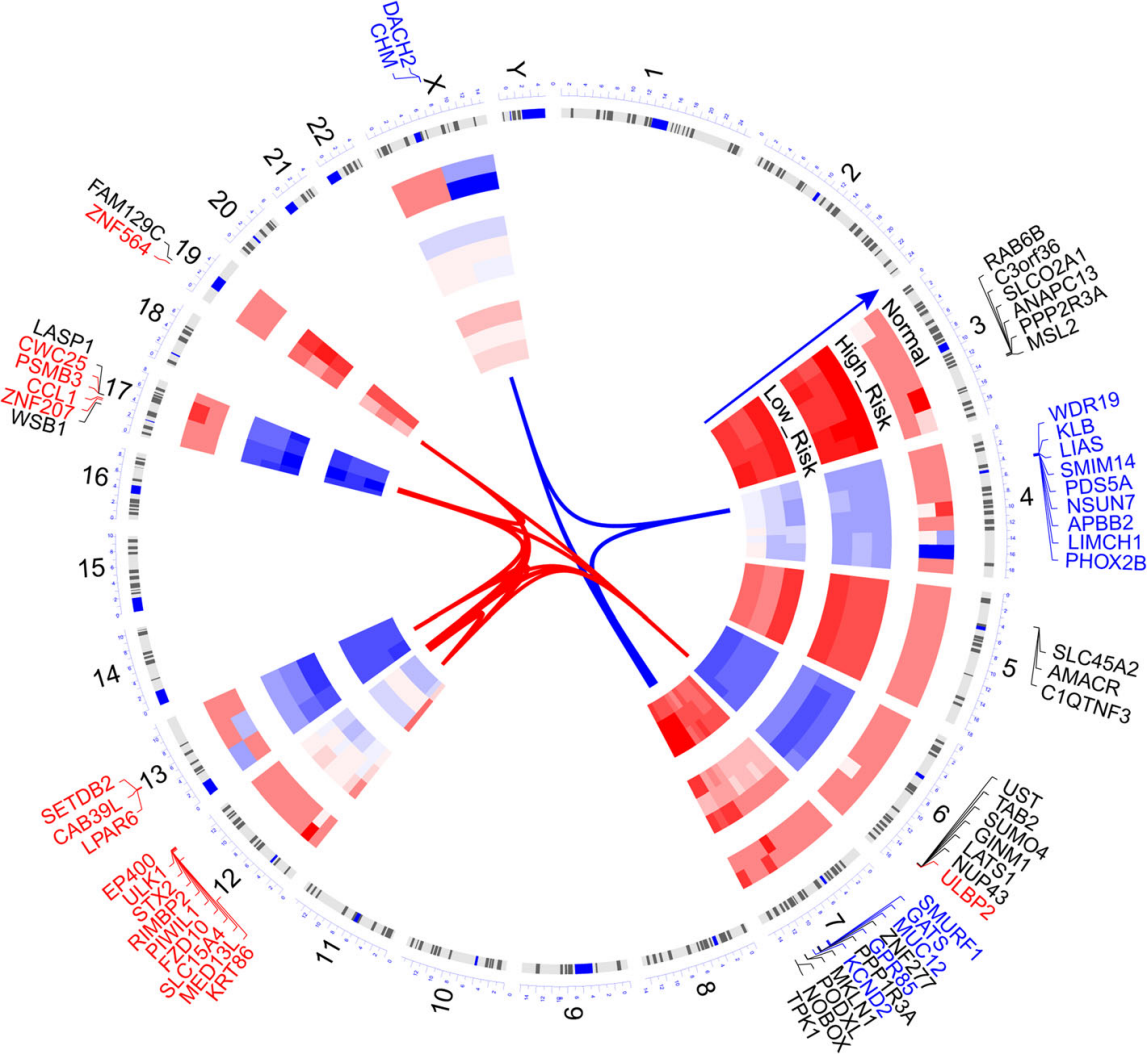
Circus plots shows the difference of copy number variations (CNV) level of genome among risk and control groups of TCGA-OV. The graph reflects location of variant genes on chromosome, red genes represent exerting amplification of copy number (> 0.1) while blue genes mean deletion (< − 0.1), and black genes reflect − 0.1 ~ 0.1 CNV level between high- and low-risk group. Figure was generated by OmicCircos (version 1.28.0)

**Fig. 8:**
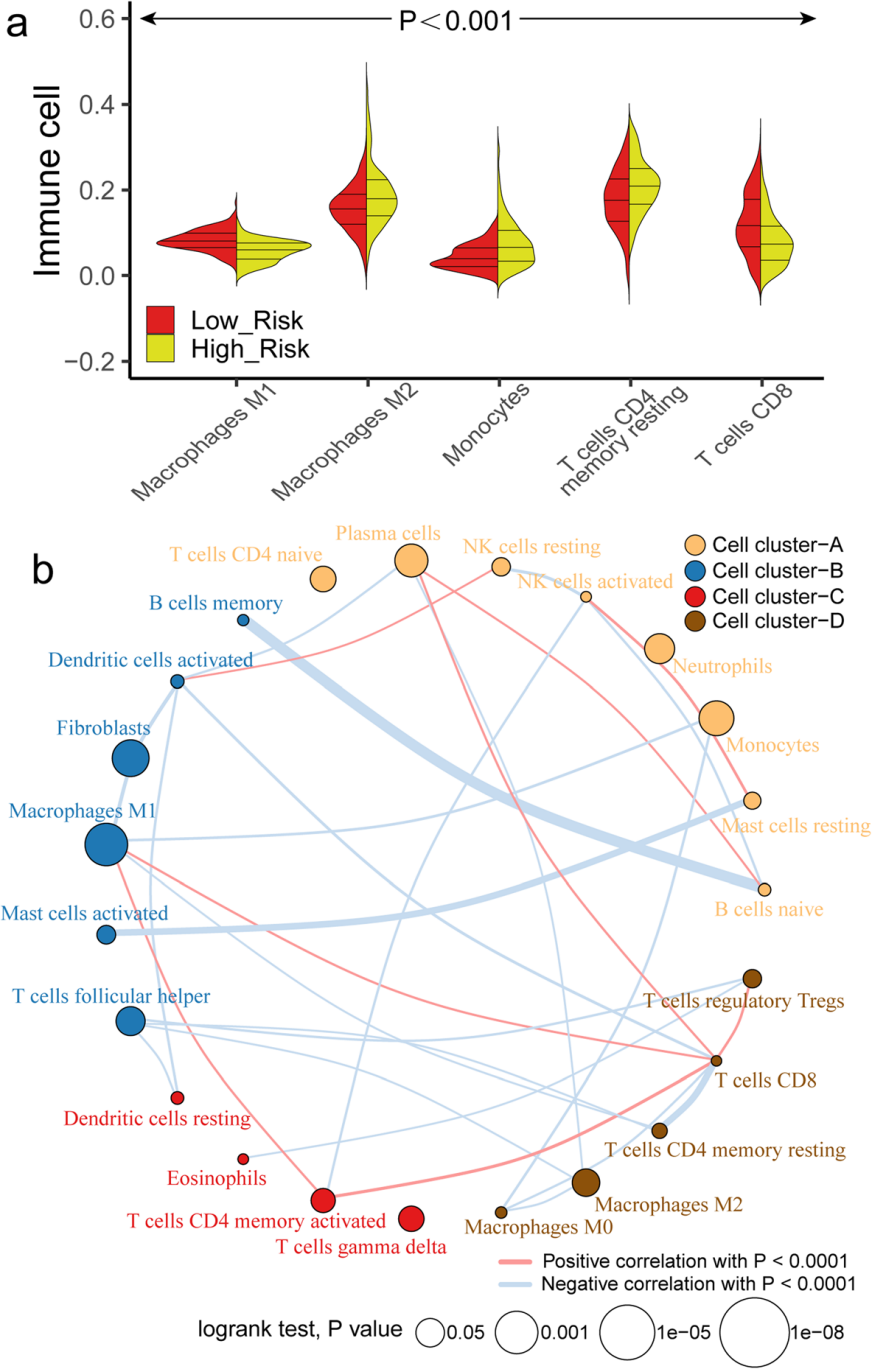
The landscape of immune infiltration in the TCGA cohort. **a** The Violin plot shows the significant difference (*P* < 0.001) of immune cell fractions between high-risk and low-risk subgroup. **b** The interaction between 22 immune cells in TCGA-OV samples. The size of circle indicated the effect of each immune cell on the prognosis, and *P* value was operated by Log-rank test. **a** was generated by ggplot2 (version 3.2.1); **b** was generated by Igraph (version 1.2.4.2)

**Fig. 9:**
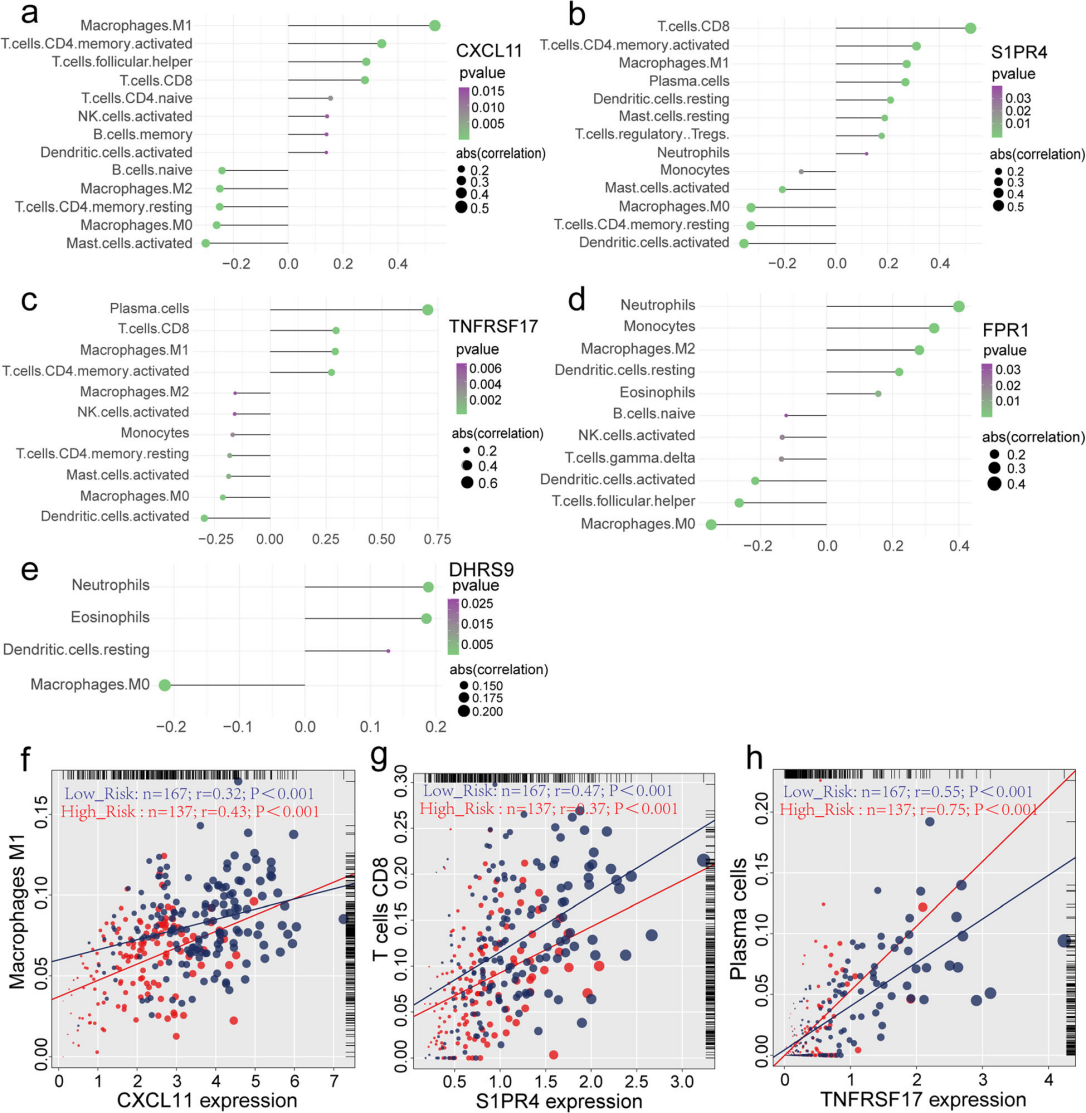
**a**-**e** Correlation between expression of 5 genes bring into classifier (CXCL11, S1PR4, TNFRSF 17, FPR1 and DHRS95) and immune cells in samples from TCGA-OV cohort (*P* < 0.05). **f**-**h** Dot plot of Pearson correlation analysis reflecting the relevance between the expression of CXCL11 and Macrophages M1, S1PR4 and T cells CD8, TNFRSF17 and Plasma cells. **a**-**e** were generated by Ggstatsplot (version 0.6.5); **f**-**h** were generated by R base graphics
