# Supplementary figures and images for: Comprehensive analysis of prognostic gene signatures based on immune infiltration of ovarian cancer
Source: BMC Cancer. 2020 Dec 7;20:1205. doi: 10.1186/s12885-020-07695-3 (PMC7720540; doi:10.1186/s12885-020-07695-3)

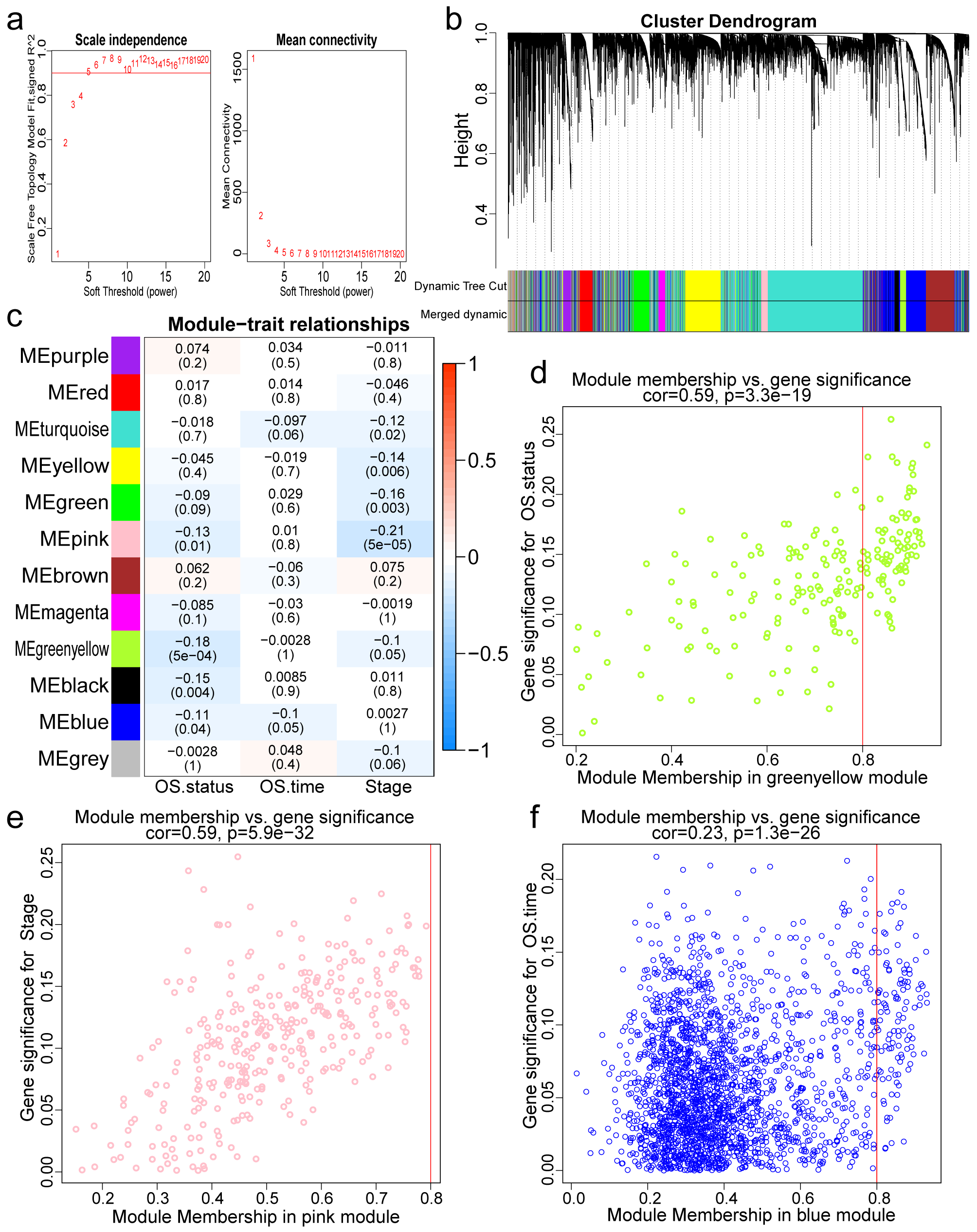

Supplement: Supplementary file 1 — Additional file 1: Figure S1. Identification of survival-related modules associated with the clinical information of ovarian cancer by weighted gene co-expression network analysis (WGCNA). (a) Analysis of the scale-free fit index and the mean connectivity for various soft-thresholding powers. (b) Clustering dendrogram of all differentially expressed genes, and each module represents a cluster of related genes and was assigned a unique color. (c) Heatmap of the correlations and differences in the modules associated with overall survival of ovarian cancer. (d) A scatter plot of gene significance for OS status vs. module membership in the green-yellow module. (e) A scatterplot of gene significance for Stage vs. module membership in the blue module. (f) A scatter plot of gene significance for OS status vs. module membership in the pink module. (d)-(f) showing a highly significant correlation between gene significance and module membership in modules. (a)-(f) were generated by WGCNA (version 1.69). [file 12885_2020_7695_MOESM1_ESM.tif]

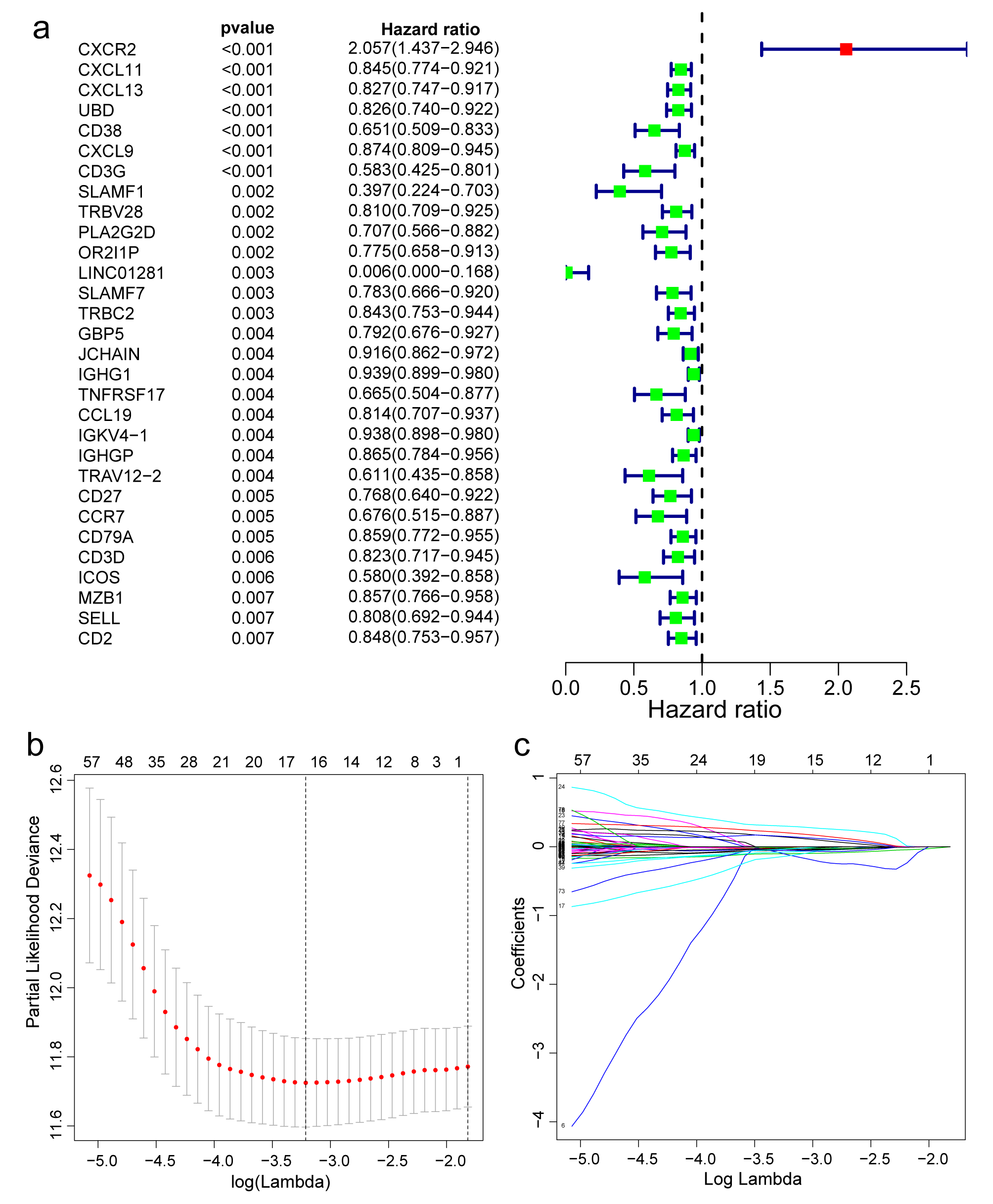

Supplement: Supplementary file 2 — Additional file 2: Figure S2. Constructing the prognostic gene classifier by the univariate cox regression and the Lasso regression analysis. (a) The top 30 most significant prognostic genes of the training set calculated by univariate cox regression. (b), (c) Determination of the number of factors through the Least absolute shrinkage and selection operator analysis (LASSO) analysis. (a) was generated by survival package (version 2.41–3); (b)-(c) were generated by glmnet package (version 3.0–1). [file 12885_2020_7695_MOESM2_ESM.tif]

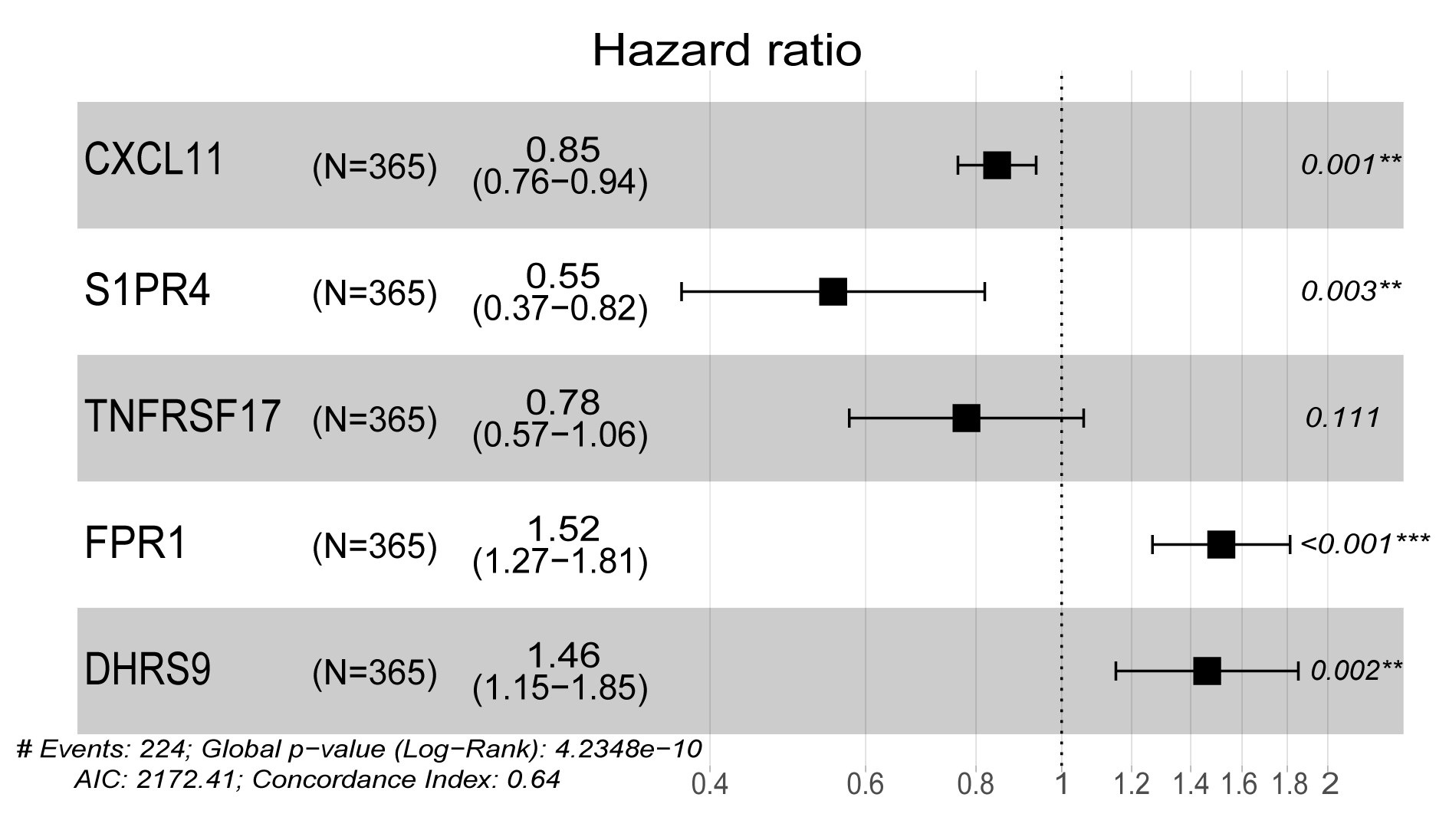

Supplement: Supplementary file 3 — Additional file 3: Figure S3. Forest plot illustrating the multivariate regression analysis results of each gene in five mRNA risk signature. The figure was generated by survminer package (version 0.4.3). [file 12885_2020_7695_MOESM3_ESM.tif]

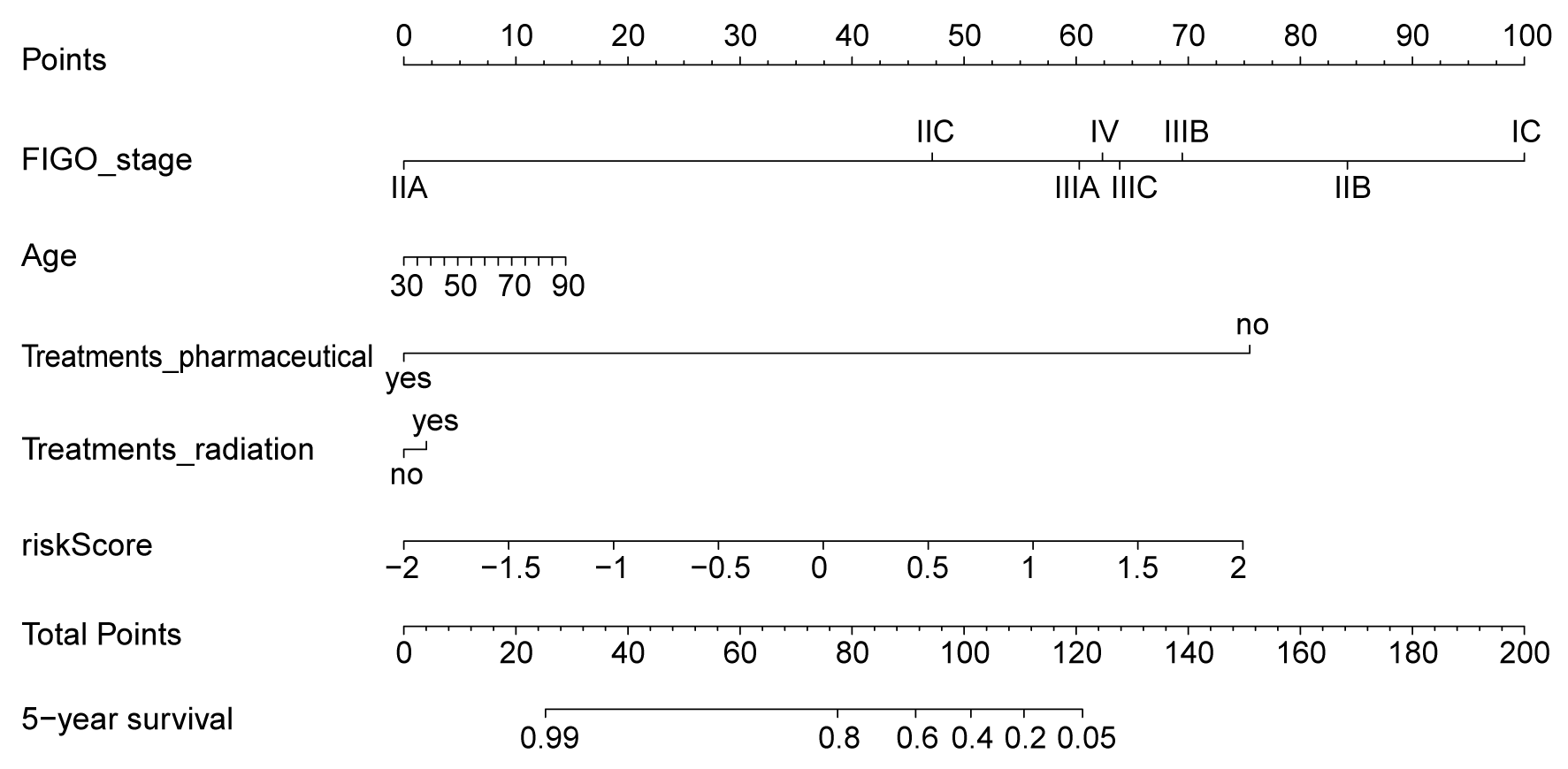

Supplement: Supplementary file 4 — Additional file 4: Figure S4. Survival nomogram of TCGA-OV samples. An individual patient’s value is located on each variable axis, and a line is drawn upward to determine the number of points received for each variable value. The sum of these numbers is located on the Total Points axis, and a line is drawn downward to the survival axes to determine the likelihood of 5-year survival. The figure was generated by Regression Modeling Strategies (version 6.0–1). [file 12885_2020_7695_MOESM4_ESM.tif]

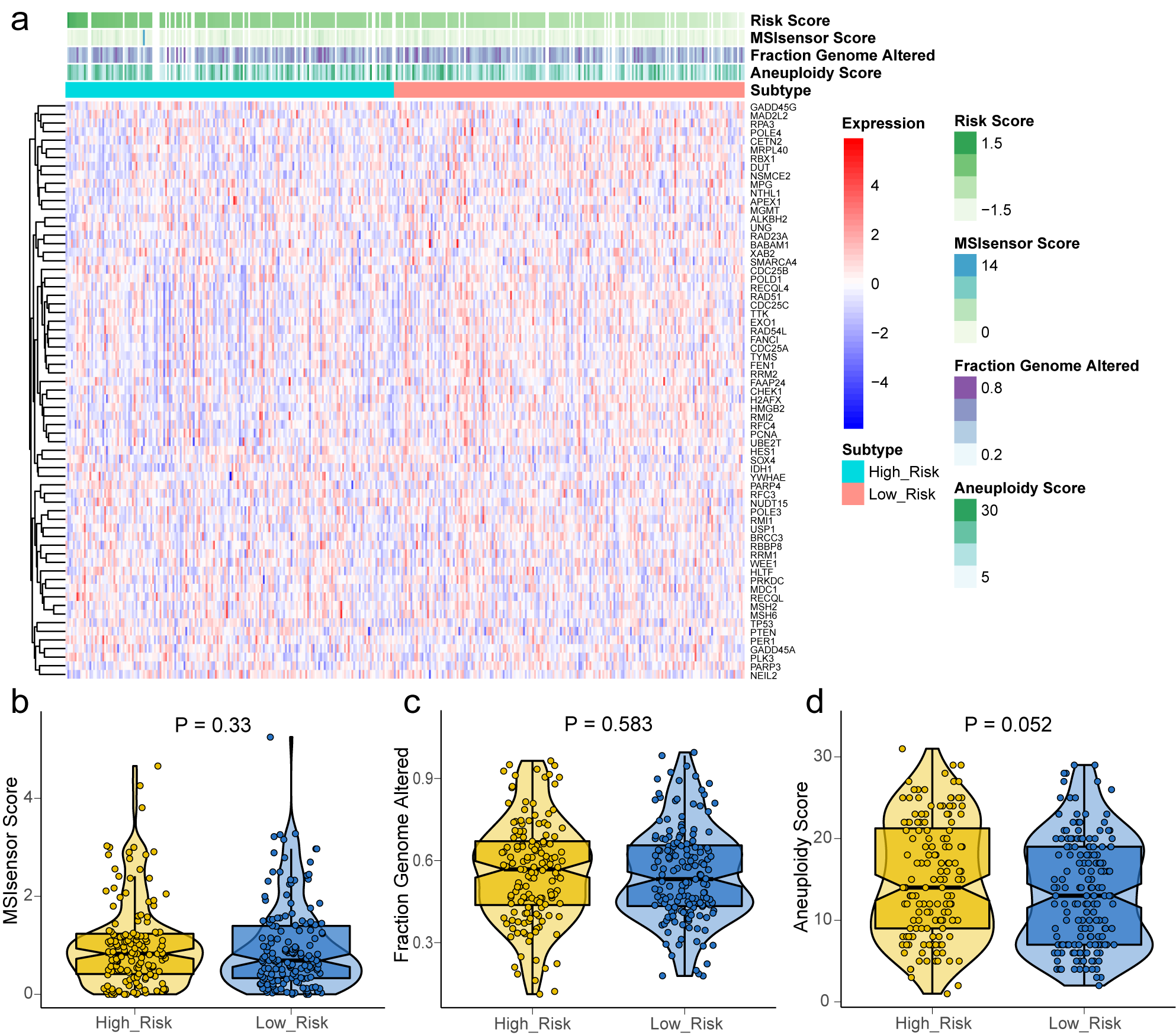

Supplement: Supplementary file 5 — Additional file 5: Figure S5. Visualization of expression of DDR-related genes and aspects of mutation status between high- and low-risk groups. (a) Heatmap showing that the expression levels of DDR-related genes were comparatively symmetrical between two subgroups. (b)-(d) The violin plots present the distribution of three features of mutation between two subgroups. (a) was generated by Pheatmap (version 1.0.12); (b)-(d) were generated by ggplot2 (version 3.2.1). [file 12885_2020_7695_MOESM5_ESM.tif]

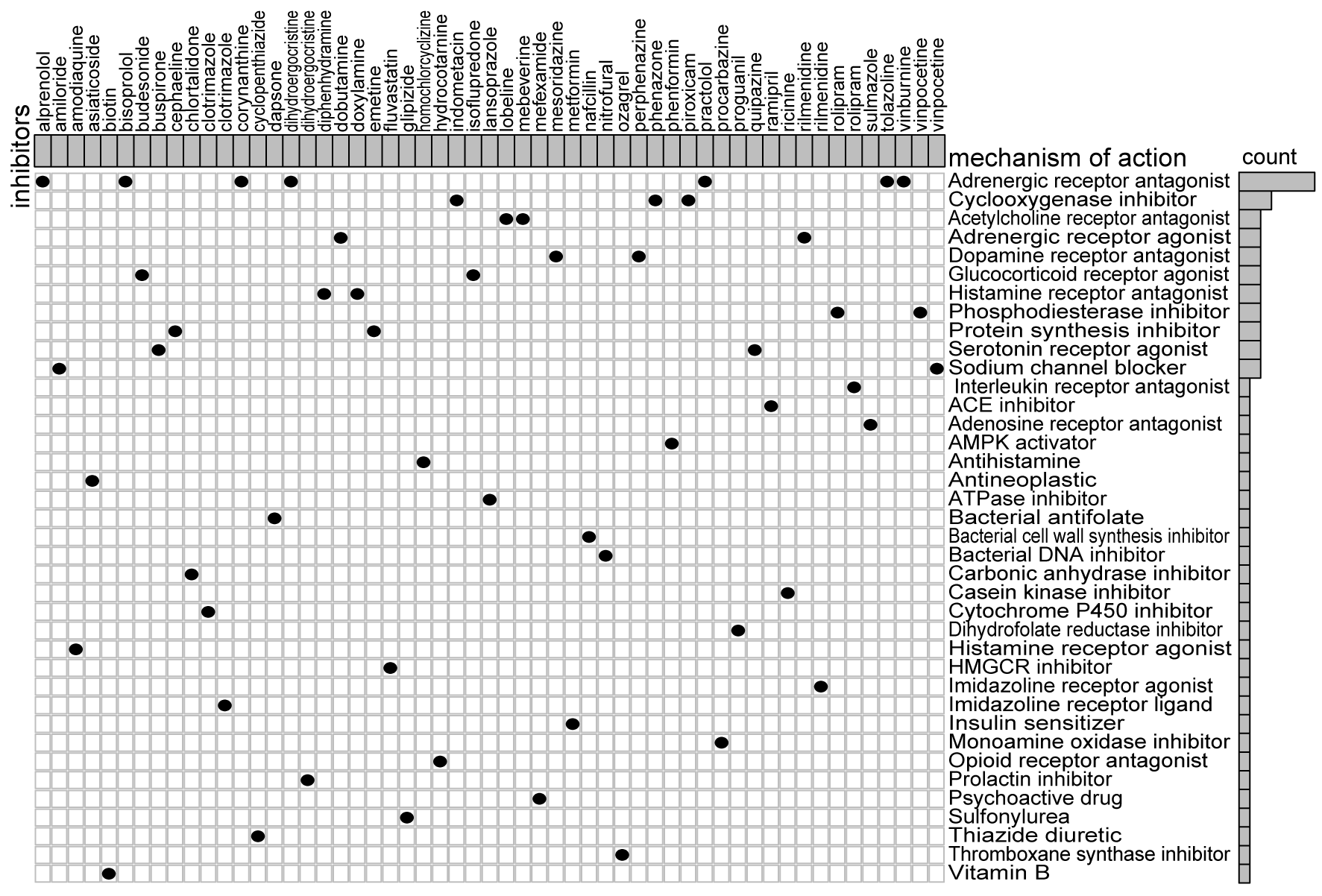

Supplement: Supplementary file 6 — Additional file 6: Figure S6. Heatmap of each compound of Connectivity Map (CMap) that shares the targeted mechanism of action. The figure was generated by ComplexHeatmap (version 2.6.2). [file 12885_2020_7695_MOESM6_ESM.tif]

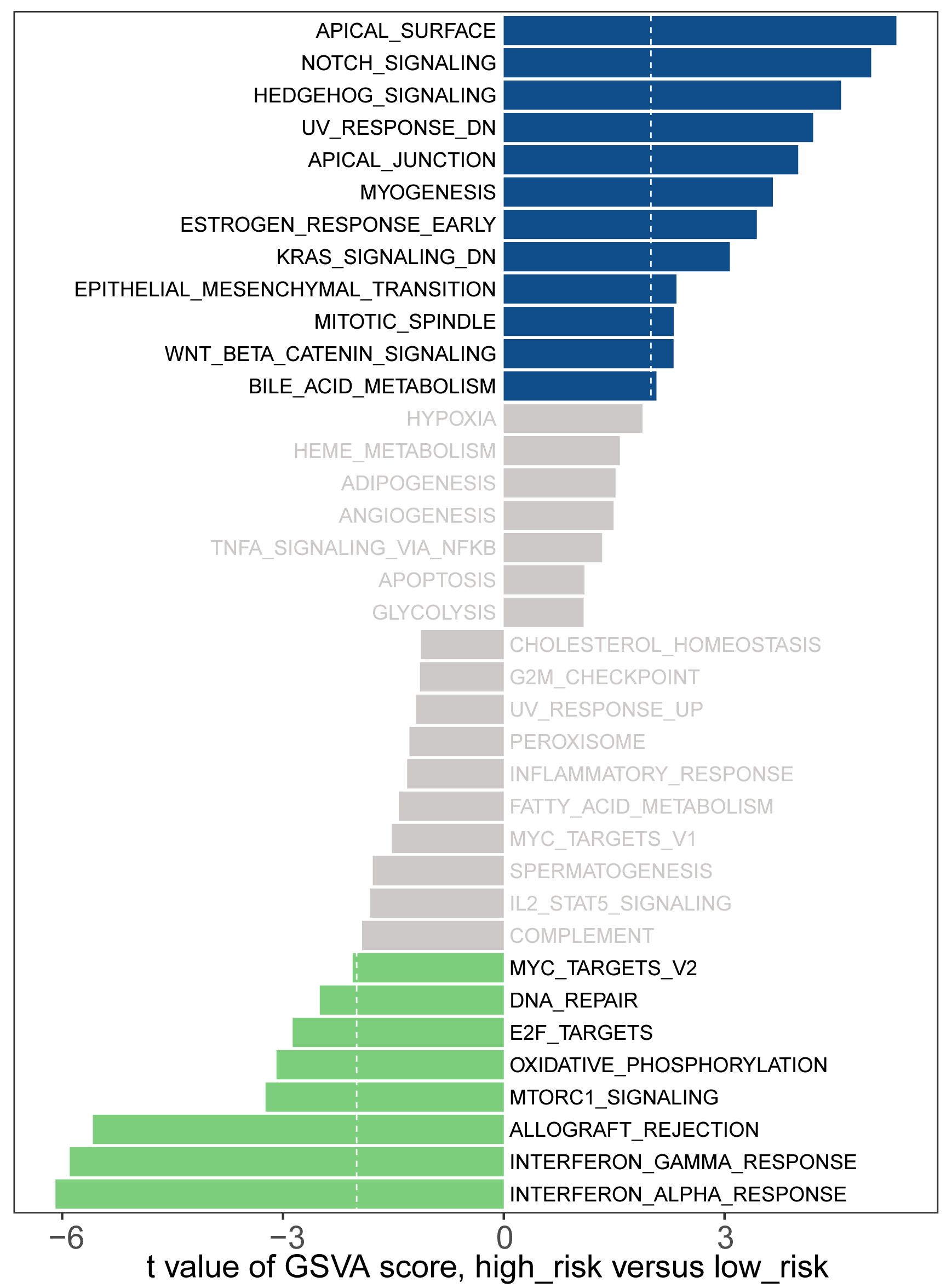

Supplement: Supplementary file 7 — Additional file 7: Figure S7. Differences in pathway activities estimated per TCGA-OV sample via gene set variation analysis (GSVA) between high-risk and low-risk subgroup. The figure was generated by ggplot2 (version 3.2.1). [file 12885_2020_7695_MOESM7_ESM.tif]
